# Genome‐Wide Association Study of Temporomandibular Disorder‐Related Pain in Finnish Populations

**DOI:** 10.1111/joor.13883

**Published:** 2024-10-31

**Authors:** J. M. Leppilahti, J. Knuutila, P. Pesonen, V. Vuollo, M. Männikkö, M. K. Karjalainen, A. L. Suominen, K. Sipilä

**Affiliations:** ^1^ Research Unit of Population Health, Faculty of Medicine University of Oulu Oulu Finland; ^2^ Northern Finland Birth Cohorts, Arctic Biobank, Infrastructure for Population Studies, Faculty of Medicine University of Oulu Oulu Finland; ^3^ Institute of Dentistry, University of Eastern Finland Kuopio Finland; ^4^ Oral and Maxillofacial Teaching Clinic Kuopio University Hospital Kuopio Finland; ^5^ Department of Public Health and Welfare National Institute for Health and Welfare (THL) Helsinki Finland; ^6^ Medical Research Center Oulu Oulu University Hospital and University of Oulu Oulu Finland

**Keywords:** birth cohort studies, chronic pain, genome‐wide association study, myalgia, public health surveillance, temporomandibular joint disorders

## Abstract

**Background:**

Temporomandibular disorders (TMD) are multifactorial musculoskeletal pain and dysfunctions in temporomandibular joints (TMJs) and masticatory muscles. Genetic factors play a role in TMD‐related pain, but only a few genome‐wide association studies (GWAS) have been conducted.

**Objective:**

The aim of this GWAS was to explore genetic factors associated with painful TMD in Finnish populations.

**Methods:**

Data from two epidemiological surveys, the Northern Finland Birth Cohort 1966 (NFBC1966) and the Health 2000 Survey in Finland, including altogether 468 cases and 6833 controls, were used. Case definition was based on pain on palpation of masticatory muscles and/or TMJs. GWASs of the whole data and stratified by sex were conducted from both cohorts using additive models, followed by meta‐analysis of the two cohorts. Replications of the previously reported TMD risk loci (rs73460075, *DMD*; rs4794106, *SGCA*; rs73271865, *SP4*; rs60249166, *RXP2*; rs1531554, *BAHCCI*; rs5862730, *OTUD4/SMAD1*; rs10092633, *SFRP1*; rs34612513, *SOX14/CLDN18*; rs878962, *TSPAN9*) were also investigated.

**Results:**

Four genome‐wide significant loci were found in sex‐stratified analysis of NFBC1966, including associations at three loci in males (rs1023114, *PRIM2*, *p* = 5 × 10^−9^; rs4244867, *ALG10*, *p* = 3 × 10^−8^; rs79841648, *ADCYAP1*, *p* = 4 × 10^−9^) and one locus in females (rs148476652, *DNER*, *p* = 4 × 10^*−9*^). However, the results could not be replicated in the Health 2000 Survey or in the meta‐analysis of these two cohorts. The previous TMD GWAS associations did not replicate in our data either.

**Conclusion:**

Several TMD pain‐associated variants were found in sex‐stratified analysis of NFBC1966, suggesting the role of neuroendocrine stress responses and central nervous system. These findings need to be confirmed in future studies.

## Graphical abstract

This study explores the genetic factors of painful temporomandibular disorders (TMD) using data from two large Finnish cohorts in a GWAS setting. The results suggest the role of genes related to nervous system development (DNER) and neuroendocrine stress response (ADCYAP1) in TMD.

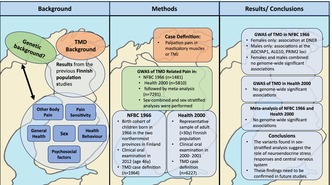

## 1 Background

Temporomandibular disorders (TMD) are a variety of dysfunctions and pain symptoms in the masticatory structures [1]. TMD‐related pain is one of the most common forms of musculoskeletal pain and linked with other pain conditions and chronic pain mechanisms [2]. The aetiology of TMD itself is complex and multifactorial. Especially, psychosocial factors [3, 4] affect the onset and chronification of TMD. TMD‐related pain is most common in premenopausal women [5], and hormonal factors may at least partly explain the sex differences [6].

TMD likely also has a genetic background. Genetic association and familial aggregation studies suggest modest evidence for the heritability of TMD pain [7]. Most early twin studies estimated that heritability of TMD is low [8, 9, 10, 11]; however, their sample sizes were low and diagnostic criteria for TMD poorly defined. In more recent twin studies with notable sample sizes and validated screening methods of TMD, heritability was estimated to be from 27% [12] to 35% [13]. Other pain complaints have given even higher heritability estimates; for example, from 35% to 68% for low back and neck pain [14].

A few risk genes for painful TMD have been presented in genetic association studies. Most of these candidate genes encode neurotransmitters or other related proteins involved in the serotonergic and catecholaminergic signalling pathways. For example, variants in *COMT* encoding catechol‐O‐methyltransferase with a role in the degradation of catecholamines such as dopamine, epinephrine, and norepinephrine have been linked with increased pain perception, possibly contributing to TMD chronification [15]. *COMT* variants have also been associated with myofascial pain and disc displacement as well as anxiety [16]. The significant role of *COMT* in TMD pathogenesis has also been confirmed in a recent systematic review and meta‐analysis [17, 18].

Genome‐wide association studies (GWAS) have given new insight into many complex multifactorial diseases. In GWAS, genetic variants associating with a specific outcome are explored agnostically and the screening of risk loci is not limited by prior knowledge of disease pathophysiology and aetiology. Only a few GWAS studies have been conducted on painful TMD. Using the US Hispanic Community Health Study/Study of Latinos with several replication cohorts, Sanders et al. (2017) discovered a few novel variants relevant to TMD pathobiological processes, including two female sex‐specific loci (near *RXP2* and *BAHCC1*) suggesting the role of inflammatory mechanisms, and two associations (near *DMD* and *SGCA*) related to the cellular structure and biomechanical properties of muscle fibers [19]. In another GWAS study by Smith et al. (2019), based on the OPPERA data set, low expression of *MRAS* gene seemed to moderate the resiliency to chronic pain; the effect was male‐speficic [20]. These results suggest that males and females develop TMD via different processes. However, the association of candidate genes (e.g., *COMT*) and TMD have so far not been replicated in GWASs. Additional genome‐wide studies are needed to explore the genetic background of TMD in different populations.

The aim of this study was to discover genetic risk factors for TMD pain using GWAS in two large Finnish population‐based samples.

## 2 Methods

Data of two population‐based studies, the Northern Finland Birth Cohort 1966 (NFBC1966) and the national Health 2000 Survey, were used in the study. The details of the two surveys are as follows.

### 2.1 NFBC1966 Study

**FIGURE 1:**
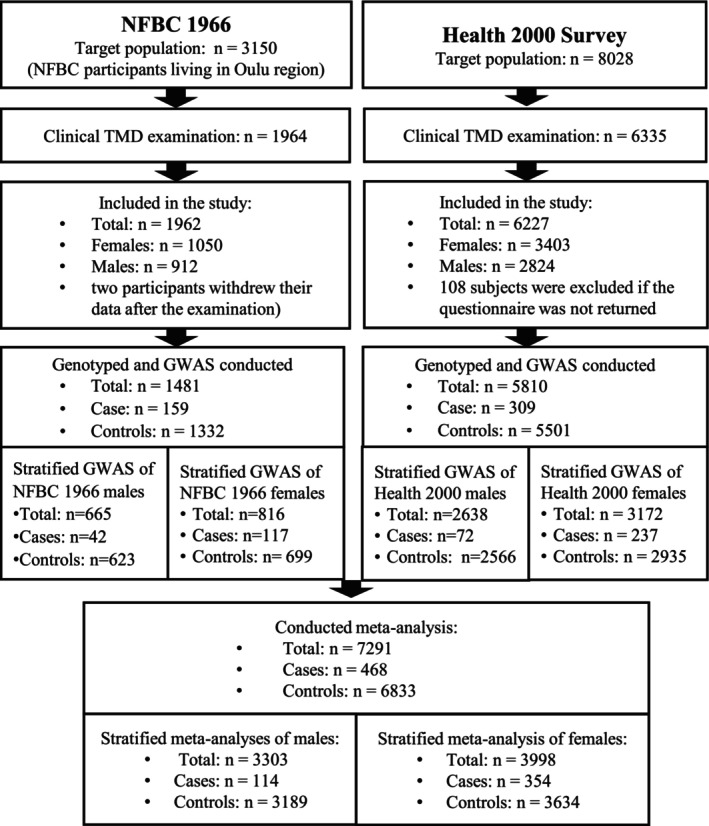
Flow chart of the study populations.

NFBC1966 is a longitudinal research programme originally consisting of 12 058 live‐born children to mothers with expected date of delivery in 1966 in the two northernmost provinces in Finland [21, 22]. Data on participants' well‐being and health status have been collected from clinical examinations and questionnaires at regular intervals. A comprehensive oral heath examination was conducted for a subsample of 1964 participants in NFBC1966 in 2012–2013 when the participants were 46 years old. Of these participants, 1481 had clinical and genotype information available and were included in this study. From the subsample with oral examination, two participants declined to take part in any genetic studies and were excluded from this study. The inclusion and exclusion of participants is described in detail in a flow chart (Figure 1). Participation in the study was voluntary, and the participants provided an informed written consent. The Ethical Committee of Northern Ostrobothnia Hospital District approved the study (74/2011; 94/2011).

Oral health examinations included TMD‐related symptom questionnaires and clinical examination which were previously described in detail by Jussila et al. [23]. Briefly, standardised clinical dental and TMD examination was conducted for participants by five calibrated dentists (examiners) at the Institute of Dentistry, University of Oulu. Interexaminer agreement was determined regularly during the study. The TMD examination followed the modified protocol of the DC/TMD presented in a symposium at the International Association for Dental Research (IADR) Conference in 2010 [24]. For the present study, pain on palpation of the masticatory muscle and TMJs were used to define the phenotype as follows: Bilateral palpation (pressure of 1.0 kg) for familiar pain of temporalis (anterior, middle and posterior regions) and masseter muscles (origin, deep and insertion regions), and around TMJ was performed. Lateral pole of the TMJ was palpated with 0.5 kg pressure. To be classified as a ‘TMD‐pain case’, presence of examination‐evoked pain in three or more temporomandibular muscles and/or TMJs was required. When neither of these findings was recorded, the subject was classified as a ‘non‐pain‐TMD control’.

### 2.2 The Health 2000 Survey

The Health 2000 Survey is a national population health examination survey including a representative sample of the adult Finnish population and was carried out by the National Institute for Health and Welfare (THL, the former National Public Health Institute of Finland, KTL) in 2000–2001. The two‐stage, stratified cluster sampling was planned by Statistics Finland. The frame of the main sample consisted of 8028 adults aged 30 years or over living in mainland Finland.

Comprehensive clinical oral examination and self‐administered questionnaire were obtained from 6227 participants. Of these participants, 5810 with a mean age of 52.9 years (SE 0.2) had clinical and genotype information available and were included in this study. A thorough description of TMD examination has been given previously by Sipilä et al. [25]. Participation in the study was voluntary, and the participants provided an informed written consent. The Ethical Committee for Epidemiology and Public Health of the Hospital District of Helsinki and Uusimaa approved the study (5/2000). Additional information about the Health 2000 Survey is available at https://thl.fi/en/web/thlfi‐en/research‐and‐expertwork/projects‐and‐programmes/health‐2000‐2011/health‐2000‐in‐brief, and the form used in the survey are available at https://thl.fi/en/web/thlfi‐en/research‐and‐expertwork/projects‐and‐programmes/health‐2000‐2011/forms/health‐2000‐forms.

Clinical TMD signs were assessed in standardised examination by five trained and calibrated dentists (Suominen‐Taipale et al., 2008). The examination included maximum mouth opening, auscultation of the TMJs and palpation of the TMJs and masticatory muscles (temporalis anterior and masseter superficialis) for pain. Palpation pain was assessed in TMJ by applying a force of approximately 0.5 kg over the immovable condyle, and in muscles with a force of approximately 1 kg. A letter scale was used to standardise the force of palpation between the examinations. The pain on joint/muscle palpation was recorded if participants (verbally) reported pain when asked or showed a protective reflex. The TMD case definition utilised in the Health 2000 Survey was similar as in the NFBC1966 study.

### 2.3 Genotyping and Statistical Analysis

Both NFBC1966 samples and Health 2000 DNA samples were genotyped using Illumina Humanhap 610 k array and imputed using SISu v2 with 2690 hcWGS and 5092 WES Finnish genomes. SNPTEST v2.5.2 and the frequentist association additive models were used for genetic analyses. Models were adjusted with the sex (in nonstratified analysis) and first four and ten principal components within the analysis of NFBC1966 and Health 2000 data, respectively. Health 2000 analyses also included adjustments for age and batch. SNPs with Hardy–Weinberg equilibrium *p* ≤ 10^−7^ were excluded, as were those with minor allele frequency < 0.05, call rate < 0.95, or imputation info score < 0.8. Meta‐analysis of the results from the two cohorts was carried out with METAL [26]; SNPs were filtered to those present in both cohorts. Painful TMD is more common in females and gene–gender interactions are probable. Therefore, stratified analyses of males and females were conducted in addition to whole data analysis. We used the traditional threshold to denote genome‐wide significance (*p* < 5 × 10^−8^), and the level of suggestive association was set at *p* < 5 × 10^−6^.

Post hoc power calculations, based on the available data in NFBC1966 and Health 2000 Survey, were conducted to estimate presumable effect sizes, taking into account the sample size of each genome‐wide discovery and meta‐analysis. Power calculations were performed utilising the web page calculator by University of Michigan (https://csg.sph.umich.edu/abecasis/cats/gas_power_calculator) derived the methods presented by Skol et al. [27]. Based on the calculation, it can be presumed that there is moderate statistical power (> 0.80) to detect common risk variants (MAF > 0.05) with at least moderate effect size (OR 2.5–3.0) in the all meta‐analysis and discovery analysis of women in Health 2000 Survey and large effect size variants (OR > 5.0–7.0) in the other discovery analysis.

### 2.4 Replication Analysis of Previously Reported Findings

Risk loci (SNPs with at least suggestive level *p* values, < 5 × 10^−6^) reported in the previous GWAS of TMD were searched from the GWAS catalogue (https://www.ebi.ac.uk/gwas/) and examined further from the original articles [19, 20, 28]. The associations of the reported risk SNPs were investigated from NFBC1966 and Health 2000 and the respective meta‐analysis, and reported separately. An association with a *p* value < 0.05 was considered significant in this replication analysis.

## 3 Results

### 3.1 Demographic Characteristics

Basic demographic characteristics and TMD case prevalences of NFBC1966 and Health 2000 Survey participants included in this study are described in Table 1. The proportion of men and women was similar, at 45% and 55%, respectively, in both cohorts. TMD prevalence was approximately double in NFBC1966 compared to the Health 2000 Survey. This trend was consistent whether all participants or those stratified by sex were analysed. The mean age of participants was slightly higher in the Health 2000 Survey, at 53 years, compared with 46 years in NFBC1966 and the age distribution was obviously wider corresponding the general population in Finland.

**TABLE 1:** Basic demographics.

	Health 2000 survey	NFBC 1966
Number of participants (%)		
All	5810	1481
Men	2638 (45.4%)	665 (44.9%)
Women	3172 (54.6%)	816 (55.1%)
Mean age in years (SD)		
All	52.9 (14.9)	46.5 (0.6)
Men	51.6 (13.9)	46.5 (0.06)
Women	54.0 (15.6)	46.5 (0.06)
Number of TMD cases (%)		
All	309 (5.3%)	159 (10.7%)
Men	72 (2.7%)	42 (6.3%)
Women	237 (7.5%)	117 (14.3%)

Abbreviations: SD, standard deviation; TMD, temporomandibular disorder case definition was based on pressure‐evoked pain at multiple sites in the masticatory muscles and the temporomandibular joint.

### 3.2 Discovery GWAS and Meta‐Analysis

Overall, no genome‐wide significant associations (*p* < 5 × 10^−8^) were found in the meta‐analysis of NFBC1966 and Health 2000 (468 cases vs. 6833 controls). The most significant association was detected for rs73022579 (*p* = 2.14 × 10^−7^) in an intron of the *PARK2* gene on Chromosome 6. Also, no significant SNPs were found in the discovery GWAS of Health 2000 data or the whole data of NFBC1966. All suggestively associated SNPs (*p* < 5 × 10^−6^) from these analyses are listed in the Supporting Information that also includes Manhattan plots illustrating the results of GWASs of NFBC1966 and Health 2000 as well as the meta‐analysis results.

**FIGURE 2:**
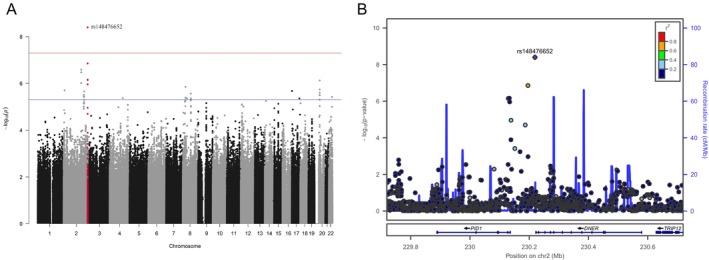
Manhattan plot (A) for the female‐specific results in discovery GWAS of NFBC1966 and respective regional association plot (B) for the significant locus near the *DNER* gene.

**FIGURE 3:**
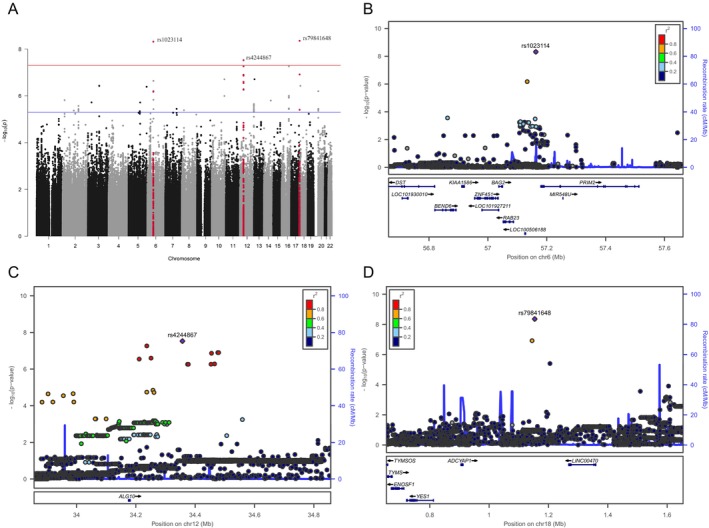
Manhattan plot (A) for the male‐specific results of discovery GWAS of NFBC1966 and respective regional association plots (B, C, D) for the significant loci near *PRIM2*, *ALG10* and *ADCYAP1* genes.

Four genome‐wide significant signals were found in the sex‐stratified analysis of the NFBC1966 data. These SNPs and related parameters are reported in Table 2. Manhattan plots and regional association plots are shown in Figures 2 and 3. In the analysis of females, one significant locus (lead SNP rs148476652, *p* = 4 × 10^−9^) was found on Chromosome 2 located downstream of *DNER* (Delta/notch like EGF repeat containing) and upstream of *PID1* (phosphotyrosine interaction domain containing 1). Association of the lead SNP was supported by a few correlated SNPs (with *p* values < 5 × 10^−6^) (Figure 2). DNER is a transmembrane protein located in dendrites and cell bodies of developing and mature CNS neurons with signal transduction‐related functions, [29] and it has been observed to play an important role in the developing central nervous system [30]. PID1 is located in cytoplasm and involved in several intracellular processes. PID1 has previously been associated with factors such as obesity and Type 2 diabetes. The mechanism in these functions is via increased lipolysis in adipose tissue and negative regulation of glucose metabolism [31, 32].

**TABLE 2:** Genome‐wide significant (*p* < 5 × 10^−8^) SNPs associated with painful TMD in NFBC1966.

Dataset	N	Chr	Position	SNP	Nearest genes	Effect allele	Effect allele frequency	OR (95%CI)	*p*
NFBC1966 Female	816	2	230 219 445	rs148476652	*DNER, PID1* (intergenic)	C	0.052	6.95 (3.64; 13.2)	4.01 × 10^−9^
NFBC1966 Male	665	6	57 162 808	rs1023114	*PRIM2*, *RAB23* (intergenic)	G	0.065	14.4 (5.90; 35.2)	4.82 × 10^−9^
NFBC1966 Male	665	12	34 357 023	rs4244867	*ALG10* (intergenic)	T	0.073	14.2 (5.55; 36.2)	2,97 × 10^−8^
NFBC1966 Male	665	18	1 153 710	rs79841648	*LINC00470, ADCYAP1* (intergenic)	T	0.060	20.7 (7.57; 57.7)	4.42 × 10^−9^

Abbreviations: Chr, chromosome; CI, confidence interval; N, number of participants; OR, odds ratio; SNP, single nucleotide polymorphism.

Among males, three significant loci were found on Chromosomes 6, 12 and 18. On Chromosome 12, the lead SNP (rs4244867, *p* = 3.0 × 10^−8^) was located near *ALG10* (ALG10 alpha‐1,2‐glucosyltransferase), and the association was supported by other correlated SNPs in the same peak (Figure 3). *ALG10* encodes a cell membrane‐associated glucosyltransferase enzyme. *ALG10* has been recently associated with progressive myoclonus epilepsies [33]. Two additional associated loci were detected on Chromosomes 6 (rs1023114, *p* = 4.8 × 10^−9^) and 18 (rs79841648, *p* = 4.4 × 10^−9^) near *RAB23* (Ras‐related protein Rab‐23, member RAS oncogene family) and *PRIM2* (DNA primase subunit 2), and near *LINC00470* and *ADCYAP1* (adenylate cyclase‐activating polypeptide 1) (Figure 3). RAB23 has been found to coordinate cranial suture morphogenesis and control suture patency via FGF10‐pERK1/2 signalling [34]. Mutations in *RAB23* have been associated with developmental disorders. *PRIM2* encodes a subunit of DNA primase, which has an important role in DNA replication. Several independent GWASs have reported significant associations at the *RAB23‐PRIM2* locus for coronary artery disease in European and Asian populations [35, 36, 37]. However, to the best of our knowledge, no obvious mechanistic link with *PRIM2* and TMD or chronic pain has been shown. *ADCYAP1* encodes a secreted propeptide that is further processed into multiple mature peptides such as a neuropeptide called pituitary adenylate cyclase‐activating polypeptide (PACAP). ADCYAP1‐related peptides stimulate adenylate cyclase, increasing cyclic adenosine monophosphate (cAMP) levels, which in turn affect transcriptional activation. ADCYAP1/PACAP has been associated with neuroendocrine stress responses; PACAP, released from sympathetic innervation of adrenal medulla, facilitates further secretion of catecholamines in response to high stress [38].

### 3.3 Replication Analyses of Previously Associated Loci

Associations at the eight previously reported suggestively associated loci were not replicated in our cohorts (see Supporting Information). The results of the replication analyses are reported in the Supporting Information.

## 4 Discussion

In this study, our aim was to conduct a GWAS of TMD‐related pain in two large Finnish population samples, that is, NFBC1966 and Health 2000 Survey, with altogether 468 cases and 6833 controls. We found four genome‐wide significant loci in GWAS of NFBC1966 data stratified by sex. However, these signals could not be replicated in GWAS of Health 2000 data or in the meta‐analysis of these two data sets.

In the analysis of females, one genome‐wide significant SNP (rs148476652, *p* = 4.01 × 10^−9^), the association of which was supported by correlated SNPs, was found on Chromosome 2 locating downstream of *DNER* and upstream of *PID1*. DNER [29] has been observed to play an important role in the developing central nervous system [30]. DNER is part of the notch signalling pathway which is considered to take part in the regulation of immune cell differentiation and functional states [39, 40]. Serum DNER levels in proteomics analysis have been associated with the development of postsurgical neuropathic pain [41]. Controversially, Giordano et al. recently reported DNER downregulation in knee osteoarthritis patients [42].

In stratified analysis of males, three significant SNPs were found. On Chromosome 12, one significant signal (rs4244867, *p* = 2.97 × 10^−8^) near *ALG10* gene was found and supported by other SNPs in the same peak (Figures 2 and 3). ALG10 has recently been associated with progressive myoclonus epilepsies [33]. However, to the best of our knowledge, no validated disease associations have been reported so far.

On Chromosomes 6 and 18, two statistically significant but more or less isolated SNPs were located near (intergenic) *RAB23* and *PRIM2* (rs1023114, *p* = 4.82 × 10^−9^) and at intron of *ADCYAP1* (rs79841648, *p* = 4.42 × 10^−9^) [39, 41, 42].

ADCYAP1 encodes a secreted propeptide that is further processed into multiple mature peptides such as the neuropeptide called pituitary adenylate cyclase‐activating polypeptide (PACAP). PACAP was first localised in the hypothalamus [43] where it is expressed most abundantly, but later it was also found in other regions in the brain and in peripheral tissues, GI tract, adrenal gland and testis [38]. Two PACAP isoforms (PACAP‐27 and ‐38) have been observed. Of these, PACAP‐38 dominates in nervous tissues. ADCYAP1/PACAP has been associated with neuroendocrine stress responses; PACAP, released from sympathetic innervation of adrenal medulla, facilitates further secretion of catecholamines in response to high stress [38]. PACAP has been associated with primary headache disorders like migraine and cluster headache in clinical studies [40, 44]. ADCYAP1 has also been connected to general nociception [45, 46]. Peripheral neural ganglia in rat TMJ have been shown to express different neuropeptides including PACAP [47] but no human studies associating ADCYAP1/PACAP with TMD have been published so far. However, there is a well‐known association between migraine and TMD [48]. There are also several studies linking genes or SNPs affecting the regulation of neural catecholamine levels with TMD. Catechol‐O‐methyltransferase (COMT) is an enzyme catalysing the degradation of catecholamines (dopamine, adrenalin, noradrenaline) and several SNPs linked to COMT have been associated with TMD and different pain conditions such as bruxism [49], anxiety [16], pain sensitivity [50, 51] and depressive symptoms‐related pain perception in TMD [50] in previous studies [17, 18]. Thus, it is reasonable to speculate that the *ADCYAP1* gene may be linked to networks of other genes affecting catecholamine levels and may have a plausible pathomechanistic link to TMD in light of previous studies.

Three TMD‐related GWA studies are included in the publicly available GWAS catalogue that lists at least suggestive level (5 × 10^−6^) associations reported in the previous GWAS of different traits (by June 28, 2023). Two out of the three studies used similar painful TMD phenotype as in this study [19, 20] with NFBC1966 data used as one of the replication cohorts. However, the significant associations found in the discovery cohorts of Northern American mixed [19] and Hispanic [19] populations could not be replicated in the NFBC1966 data. The aim of this study was to study painful TMD in more homogenous Finnish population utilising the Health 2000 survey and to conduct a meta‐analysis of these two population studies. The third GWAS work, reported in the GWAS catalogue, explored the genetic background of TMJ bony changes indicating osteoarthritis. The present study found no direct replication of the candidate risk loci reported in these three GWA studies (Supporting Information).

The main strengths of this study are the GWAS approach and the data sets used. Not many studies have utilised the GWAS approach when examining TMD, especially in European populations. Both our datasets consisted of Finnish subjects and formed a genetically homogeneous entity, offering a great opportunity to examine the possible genetic risk factors for TMD. However, the data sets can also be seen as a limitation to the study, as the total number of subjects with genetic data was small for a genome‐wide study and limited to explore common (MAF > 0.05) moderate or large effect size variants (OR > 2.5).

Similar methods were used to define TMD cases in both cohorts, based on pressure‐evoked pain. In the Health 2000 study, insufficient clinical information prevented exact DC/TMD diagnoses. In NFBC1966, exact diagnoses were available, but the number of cases was too low for an independent GWAS, so a proxy phenotype was created to match the data in Health 2000 Survey. The case frequency of this phenotype was higher in NFBC1966 (10.7%) compared to Health 2000 Survey (5.3%) (Table 1).

Previously reported prevalence rates of muscle pain at a single site were similar between NFBC1966 and Health 2000 (11% vs. 13%) [23, 25]. However, TMJ pain was more common in NFBC1966 (10.4%) than in Health 2000 (3.8%), partly explaining the higher TMD prevalence in NFBC1966 [23, 25]. Age differences also contributed: NFBC1966 participants were all 46 years old, while Health 2000 included a wider age range from 30 to 80 years, with a mean age at 52.9 (SD 14.9) years [52]. This wider age range in Health 2000 likely explains the lower prevalence of pressure‐evoked TMD pain in multiple sites. Basic demographics and risk factors have been published previously for both cohorts, showing that general health problems and female gender are associated with TMD signs. Both cohorts had similar sex distributions (55% female) and self‐reported health conditions (60% reported good health) [25, 53]. Gene–lifestyle interaction analysis was beyond this study's scope due to sample size limitations, but different predisposing factors for TMD may exist across different ages and cohorts, potentially confounding meta‐analysis results [52].

## 5 Conclusion

In conclusion, promising genome‐wide significant associations of TMD pain were found at the *ADCYAP1* and *DNER* loci in sex‐stratified analysis of NFBC1966. The number of cases and statistical power in the stratified analysis was low in the independent analysis of NFB1966 and these findings need to be replicated and validated in future studies.

## Conflicts of Interest

The authors declare no conflicts of interest.

## Supporting information


**Data S1.** Tables reporting genome‐wide suggestive (*p* < 5 × 10^−6^) SNPs associated with painful TMD in females, males and all participants in NFBC1966, Health 2000 survey and the meta‐analysis and the respective Manhattan plots illustrating the GWAS results.

## Data Availability

NFBC data are available from the University of Oulu, Infrastructure for Population Studies. Permission to use the data can be applied for research purposes via an electronic material request portal. In the use of data, we follow the EU general data protection regulation (679/2016) and the Finnish Data Protection Act. The use of personal data is based on a cohort participant's written informed consent in their latest follow‐up study, which may cause limitations to its use. Please, contact the NFBC project center (NFBCprojectcenter@oulu.fi) and visit the cohort website (www.oulu.fi/nfbc) for more information.

## References

[1] T. List and R. H. Jensen , “Temporomandibular Disorders: Old Ideas and New Concepts,” Cephalalgia 37, no. 7 (2017): 692–704, 10.1177/0333102416686302. 28068790

[2] R. Ohrbach , R. B. Fillingim , F. Mulkey , et al., “Clinical Findings and Pain Symptoms as Potential Risk Factors for Chronic TMD: Descriptive Data and Empirically Identified Domains From the OPPERA Case‐Control Study,” Journal of Pain 12, no. 11 Suppl (2011): T27–T45, 10.1016/j.jpain.2011.09.001. 22074750 PMC3443556

[3] T. I. Suvinen , P. C. Reade , P. Kemppainen , M. Könönen , and S. F. Dworkin , “Review of Aetiological Concepts of Temporomandibular Pain Disorders: Towards a Biopsychosocial Model for Integration of Physical Disorder Factors With Psychological and Psychosocial Illness Impact Factors,” European Journal of Pain 9, no. 6 (2005): 613–633, 10.1016/j.ejpain.2005.01.012. 15978854

[4] G. C. Felin , C. V. D. C. Tagliari , B. A. Agostini , and K. Collares , “Prevalence of Psychological Disorders in Patients With Temporomandibular Disorders: A Systematic Review and Meta‐Analysis,” Journal of Prosthetic Dentistry 13 (2022): 392–401, 10.1016/j.prosdent.2022.08.002. 36114016

[5] N. Yekkalam and A. Wänman , “Prevalence of Signs and Symptoms Indicative of Temporomandibular Disorders and Headaches in 35‐, 50‐, 65‐ and 75‐Year‐Olds Living in Västerbotten, Sweden,” Acta Odontologica Scandinavica 72, no. 6 (2014): 458–465, 10.3109/00016357.2013.860620. 24417523

[6] M. P. Warren and J. L. Fried , “Temporomandibular Disorders and Hormones in Women,” Cells, Tissues, Organs 169, no. 3 (2001): 187–192, 10.1159/000047881. 11455113

[7] C. M. Visscher and F. Lobbezoo , “TMD Pain Is Partly Heritable. A Systematic Review of Family Studies and Genetic Association Studies,” Journal of Oral Rehabilitation 42, no. 5 (2015): 386–399, 10.1111/joor.12263. 25523980

[8] A. Heiberg , B. Helöe , A. N. Heiberg , et al., “Myofascial Pain Dysfunction (MPD) Syndrome in Twins,” Community Dentistry and Oral Epidemiology 8, no. 8 (1980): 434–436, 10.1111/j.1600-0528.1980.tb01323.x. 6942960

[9] K. G. Raphael , J. J. Marbach , R. M. Gallagher , and B. P. Dohrenwend , “Myofascial TMD Does Not Run in Families,” Pain 80, no. 1–2 (1999): 15–22, 10.1016/s0304-3959(98)00180-8. 10204713

[10] B. S. Michalowicz , B. L. Pihlstrom , J. S. Hodges , and T. J. Bouchard , “No Heritability of Temporomandibular Joint Signs and Symptoms,” Journal of Dental Research 79, no. 8 (2000): 1573–1578, 10.1177/00220345000790080801. 11023277

[11] Y. Matsuka , C. Nagamatsu , S. Itoh , et al., “Comparison of Inter‐Twin Concordance in Symptoms of Temporomandibular Disorders: A Preliminary Investigation in an Adolescent Twin Population,” Cranio: The Journal of Craniomandibular Practice 25, no. 1 (2007): 23–29, 10.1179/crn.2007.005. 17304914

[12] O. Plesh , S. H. Adams , and S. A. Gansky , “Self‐Reported Comorbid Pains in Severe Headaches or Migraines in a US National Sample,” Headache 52, no. 6 (2012): 946–956, 10.1111/j.1526-4610.2012.02155.x. 22553936 PMC3370154

[13] C. M. Visscher , M. J. Schouten , L. Ligthart , C. M. van Houtem , A. de Jongh , and D. I. Boomsma , “Shared Genetics of Temporomandibular Disorder Pain and Neck Pain: Results of a Twin Study,” Journal of Oral & Facial Pain and Headache 32, no. 2 (2018): 107–112, 10.11607/ofph.2016. 29509827

[14] T. Nyman , M. Mulder , A. Iliadou , M. Svartengren , and C. Wiktorin , “High Heritability for Concurrent Low Back and Neck‐Shoulder Pain: A Study of Twins,” Spine (Phila Pa 1976) 36, no. 22 (2011): E1469–E1476, 10.1097/BRS.0b013e3181e2c878. 21192295

[15] S. B. Smith , D. W. Maixner , J. D. Greenspan , et al., “Potential Genetic Risk Factors for Chronic TMD: Genetic Associations From the OPPERA Case Control Study,” Journal of Pain 12, no. 11 Suppl (2011): T92–T101, 10.1016/j.jpain.2011.08.005. 22074755 PMC3268684

[16] J. A. Brancher , P. P. Spada , M. N. Meger , et al., “The Association of Genetic Polymorphisms in Serotonin Transporter and Catechol‐O‐Methyltransferase on Temporomandibular Disorders and Anxiety in Adolescents,” Journal of Oral Rehabilitation 46, no. 7 (2019): 597–604, 10.1111/joor.12783. 30811655

[17] J. A. Brancher , F. M. P. Bertoli , B. Michels , et al., “Is Catechol‐O‐Methyltransferase Gene Associated With Temporomandibular Disorders? A Systematic Review and Meta‐Analysis,” International Journal of Paediatric Dentistry 31, no. 1 (2021): 152–163, 10.1111/ipd.12721. 32961632

[18] D. Cruz , F. Monteiro , M. Paço , et al., “Genetic Overlap Between Temporomandibular Disorders and Primary Headaches: A Systematic Review,” Japanese Dental Science Review 58 (2022): 69–88, 10.1016/j.jdsr.2022.02.002. 35242249 PMC8881721

[19] A. E. Sanders , D. Jain , T. Sofer , et al., “GWAS Identifies New Loci for Painful Temporomandibular Disorder: Hispanic Community Health Study/Study of Latinos,” Journal of Dental Research 96, no. 3 (2017): 277–284, 10.1177/0022034516686562. 28081371 PMC5298397

[20] S. B. Smith , M. Parisien , E. Bair , et al., “Genome‐Wide Association Reveals Contribution of MRAS to Painful Temporomandibular Disorder in Males,” Pain 160, no. 3 (2019): 579–591, 10.1097/j.pain.0000000000001438. 30431558 PMC6377338

[21] T. Nordström , J. Miettunen , J. Auvinen , et al., “Cohort Profile: 46 Years of Follow‐Up of the Northern Finland Birth Cohort 1966 (NFBC1966),” International Journal of Epidemiology 50, no. 6 (2022): 1786–1787j, 10.1093/ice/dyab109. 34999878 PMC8743124

[22] University of Oulu , “Northern Finland Birth Cohort,” 1966 University of Oulu, http://urn.fi/urn:nbn:fi:att:bc1e5408‐980e‐4a62‐b899‐43bec3755243.

[23] P. Jussila , H. Kiviahde , R. Näpänkangas , et al., “Prevalence of Temporomandibular Disorders in the Northern Finland Birth Cohort 1996,” Journal of Oral & Facial Pain and Headache 31, no. 2 (2017): 159–164, 10.11607/ofph.1773. 28437513

[24] R. Ohrbach , T. List , J. P. Goulet , and P. Svensson , “Recommendations From the International Consensus Workshop: Convergence on an Orofacial Pain Taxonomy,” Journal of Oral Rehabilitation 37, no. 10 (2010): 807–812, 10.1111/j.1365-2842.2010.02088.x. 20374436

[25] K. Sipilä , A. L. Suominen , P. Alanen , M. Heliövaara , P. Tiittanen , and M. Könönen , “Association of Clinical Findings of Temporomandibular Disorders (TMD) With Self‐Reported Musculoskeletal Pains,” European Journal of Pain 15, no. 10 (2011): 1061–1067, 10.1016/j.ejpain.2011.05.001. 21664847

[26] C. J. Willer , Y. Li , and G. R. Abecasis , “METAL: Fast and Efficient Meta‐Analysis of Genome wide anssociation Scans,” Bioinformatics 26, no. 17 (2010): 2190–2191, 10.1093/bioinformatics/btq340. 20616382 PMC2922887

[27] A. D. Skol , L. J. Scott , G. R. Abecasis , and M. Boehnke , “Joint Analysis Is More Efficient Than Replication‐Based Analysis for Two‐Stage Genome‐Wide Association Studies,” Nature Genetics 38, no. 2 (2006): 209–213, 10.1038/ng1706. 16415888

[28] T. Yamaguchi , H. Nakaoka , K. Yamamoto , et al., “Genome‐Wide Association Study of Degenerative Bony Changes of the Temporomandibular Joint,” Oral Diseases 20, no. 4 (2014): 409–415, 10.1111/odi.12141. 23746317

[29] M. Eiraku , Y. Hirata , H. Takeshima , T. Hirano , and M. Kengaku , “Delta/Notch‐Like Epidermal Growth Factor (EGF)‐related Receptor, a Novel EGF‐Like Repeat‐Containing Protein Targeted to Dendrites of Developing and Adult Central Nervous System Neurons,” Journal of Biological Chemistry 277, no. 28 (2002): 25400–25407, 10.1074/jbc.M110793200. 11950833

[30] S. Y. Saito and H. Takeshima , “DNER as Key Molecule for Cerebellar Maturation,” Cerebellum 5, no. 3 (2006): 227–231, 10.1080/14734220600632564. 16997755

[31] A. W. Fischer , K. Albers , C. Schlein , et al., “PID1 Regulates Insulin‐Dependent Glucose Uptake by Controlling Intracellular Sorting of GLUT4‐Storage Vesicles,” Biochimica et Biophysica Acta ‐ Molecular Basis of Disease 1865, no. 6 (2019): 1592–1603, 10.1016/j.bbadis.2019.03.010. 30904610 PMC6624118

[32] C. Yin , W. H. Liu , Y. Liu , L. Wang , and Y. Xiao , “PID1 Alters the Antilipolytic Action of Insulin and Increases Lipolysis via Inhibition of AKT/PKA Pathway Activation,” PLoS One 14, no. 4 (2019): e0214606, 10.1371/journal.pone.0214606. 30990811 PMC6467375

[33] C. Courage , K. L. Oliver , E. J. Park , et al., “Progressive Myoclonus Epilepsies‐Residual Unsolved Cases Have Marked Genetic Heterogeneity Including Dolichol‐Dependent Protein Glycosylation Pathway Genes,” American Journal of Human Genetics 108, no. 4 (2021): 722–738, 10.1016/j.ajhg.2021.03.013. 33798445 PMC8059372

[34] M. R. Hasan , M. Takatalo , H. Ma , R. Rice , T. Mustonen , and D. P. Rice , “RAB23 Coordinates Early Osteogenesis by Repressing FGF10‐pERK1/2 and GLI1,” eLife 9 (2020): 9, 10.7554/eLife.55829. PMC7423339 32662771

[35] S. Sakaue , M. Kanai , Y. Tanigawa , et al., “A Cross‐Population Atlas of Genetic Associations for 220 Human Phenotypes,” Nature Genetics 53, no. 10 (2021): 1415–1424, 10.1038/s41588-021-00931-x. 34594039 PMC12208603

[36] G. Temprano‐Sagrera , C. M. Sitlani , W. P. Bone , et al., “Multi‐Phenotype Analyses of Hemostatic Traits With Cardiovascular Events Reveal Novel Genetic Associations,” Journal of Thrombosis and Haemostasis 20, no. 6 (2022): 1331–1349, 10.1111/jth.15698. 35285134 PMC9314075

[37] J. A. Hartiala , Y. Han , Q. Jia , et al., “Genome‐Wide Analysis Identifies Novel Susceptibility Loci for Myocardial Infarction,” European Heart Journal 42, no. 9 (2021): 919–933, 10.1093/eurheartj/ehaa1040. 33532862 PMC7936531

[38] A. J. Harmar , J. Fahrenkrug , I. Gozes , et al., “Pharmacology and Functions of Receptors for Vasoactive Intestinal Peptide and Pituitary Adenylate Cyclase‐Activating Polypeptide: IUPHAR Review 1,” British Journal of Pharmacology 166, no. 1 (2012): 4–17, 10.1111/j.1476-5381.2012.01871.x. 22289055 PMC3415633

[39] B. Zhou , W. Lin , Y. Long , et al., “Notch Signaling Pathway: Architecture, Disease, and Therapeutics,” Signal Transduction and Targeted Therapy 7, no. 1 (2022): 95, 10.1038/s41392-022-00934-y. 35332121 PMC8948217

[40] H. W. Schytz , S. Birk , T. Wienecke , C. Kruuse , J. Olesen , and M. Ashina , “PACAP38 Induces Migraine‐Like Attacks in Patients With Migraine Without Aura,” Brain 132, no. Pt 1 (2009): 16–25, 10.1093/brain/awn307. 19052139

[41] J. Lötsch , L. Mustonen , H. Harno , and E. Kalso , “Machine‐Learning Analysis of Serum Proteomics in Neuropathic Pain After Nerve Injury in Breast Cancer Surgery Points at Chemokine Signaling via SIRT2 Regulation,” International Journal of Molecular Sciences 23, no. 7 (2022): 3488, 10.3390/ijms23073488. PMC8998280 35408848

[42] R. Giordano , K. K. Petersen , H. H. Andersen , O. Simonsen , and L. Arendt‐Nielsen , “Serum Inflammatory Markers in Patients With Knee Osteoarthritis: A Proteomic Approach,” Clinical Journal of Pain 36, no. 4 (2020): 229–237, 10.1097/AJP.0000000000000804. 31977377

[43] A. Miyata , A. Arimura , R. R. Dahl , et al., “Isolation of a Novel 38 Residue‐Hypothalamic Polypeptide Which Stimulates Adenylate Cyclase in Pituitary Cells,” Biochemical and Biophysical Research Communications 164, no. 1 (1989): 567–574, 10.1016/0006-291x(89)91757-9. 2803320

[44] L. Edvinsson , J. Tajti , L. Szalárdy , and L. Vécsei , “PACAP and Its Role in Primary Headaches,” Journal of Headache and Pain 19, no. 1 (2018): 21, 10.1186/s10194-018-0852-4. 29523978 PMC5845082

[45] J. Tajti , B. Tuka , B. Botz , Z. Helyes , and L. Vecsei , “Role of Pituitary Adenylate Cyclase‐Activating Polypeptide in Nociception and Migraine,” CNS & Neurological Disorders Drug Targets 14, no. 4 (2015): 540–553, 10.2174/1871527314666150429114234. 25921738

[46] T. Dickinson and S. M. Fleetwood‐Walker , “VIP and PACAP: Very Important in Pain?,” Trends in Pharmacological Sciences 20, no. 8 (1999): 324–329, 10.1016/s0165-6147(99)01340-1. 10431211

[47] R. Uddman , T. Grunditz , J. Kato , and F. Sundler , “Distribution and Origin of Nerve Fibers in the Rat Temporomandibular Joint Capsule,” Anatomy and Embryology 197, no. 4 (1998): 273–282, 10.1007/s004290050137. 9565320

[48] J. Ashraf , N. Zaproudina , A. L. Suominen , K. Sipilä , M. Närhi , and T. Saxlin , “Association Between Temporomandibular Disorders Pain and Migraine: Results of the Health 2000 Survey,” Journal of Oral & Facial Pain and Headache 33, no. 4 (2019): 399–407, 10.11607/ofph.2213. 31247056

[49] A. R. Vieira , R. Scariot , J. T. Gerber , et al., “Bruxism Throughout the Lifespan and Variants in MMP2, MMP9 and COMT,” Journal of Personalized Medicine 10, no. 2 (2020): 44, 10.3390/jpm10020044. PMC7354525 32471213

[50] C. Schwahn , H. J. Grabe , M. zu Schwabedissen , et al., “The Effect of Catechol‐O‐Methyltransferase Polymorphisms on Pain Is Modified by Depressive Symptoms,” European Journal of Pain 16, no. 6 (2012): 878–889, 10.1002/j.1532-2149.2011.00067.x. 22337325

[51] L. L. Bonato , V. Quinelato , P. C. de Felipe Cordeiro , et al., “Polymorphisms in COMT, ADRB2 and HTR1A Genes Are Associated With Temporomandibular Disorders in Individuals With Other Arthralgias,” Cranio 39, no. 4 (2021): 351–361, 10.1080/08869634.2019.1632406. 31264537

[52] T. Rutkiewicz , M. Könönen , L. Suominen‐Taipale , A. Nordblad , and P. Alanen , “Occurrence of Clinical Signs of Temporomandibular Disorders in Adult Finns,” Journal of Orofacial Pain 20, no. 3 (2006): 208–217. 16913430

[53] P. Jussila , J. Knuutila , S. Salmela , et al., “Association of Risk Factors With Temporomandibular Disorders in the Northern Finland Birth Cohort 1966,” Acta Odontologica Scandinavica 76, no. 7 (2018): 525–529, 10.1080/00016357.2018.1479769. 29916756

