# Supplementary material for: Genome‐Wide Association Study of Temporomandibular Disorder‐Related Pain in Finnish Populations
Source: J Oral Rehabil. 2024 Oct 31;52(2):151–9. doi: 10.1111/joor.13883 (PMC11740273; doi:10.1111/joor.13883)
Supplement: Supplementary file 1 — Data S1. Tables reporting genome‐wide suggestive (p < 5 × 10^−6^) SNPs associated with painful TMD in females, males and all participants in NFBC1966, Health 2000 survey and the meta‐analysis and the respective Manhattan plots illustrating the GWAS results. [file JOOR-52-151-s001.docx]

**Supplementary file for the original article – “Genome-wide Association Study of Temporomandibular Disorder Related Pain in Finnish Population”**

**Table 1. Genome-wide suggestive (*p* < 5 x 10^-6^) SNPs associated with painful TMD in all participants (N = 1481) of NFBC 1966 study.**

| **Chr** | **Position** | **SNP** | **Effect allele** | **Effect allele frequency** | **OR** | **95% CI lower** | **95% CI upper** | **p-value** |
| --- | --- | --- | --- | --- | --- | --- | --- | --- |
| 1 | 212800886 | rs10863983 | G | 0,75 | 0,52 | 0,40 | 0,68 | 1,82E-06 |
| 2 | 85992512 | rs4832009 | C | 0,82 | 2,08 | 1,53 | 2,83 | 3,48E-06 |
| 2 | 85993077 | rs12475263 | C | 0,82 | 2,08 | 1,52 | 2,83 | 3,52E-06 |
| 2 | 230219445 | rs148476652 | C | 0,05 | 4,30 | 2,47 | 7,49 | 2,62E-07 |
| 6 | 28323968 | rs2079705 | A | 0,08 | 3,16 | 2,04 | 4,88 | 2,31E-07 |
| 6 | 28326535 | rs144931476 | A | 0,07 | 2,92 | 1,85 | 4,61 | 4,50E-06 |
| 6 | 28503137 | rs138510431 | T | 0,08 | 2,94 | 1,86 | 4,65 | 4,03E-06 |
| 8 | 41817704 | rs141641517 | C | 0,07 | 3,57 | 2,22 | 5,74 | 1,43E-07 |
| 8 | 41947327 | rs146966082 | C | 0,06 | 3,37 | 2,01 | 5,65 | 3,99E-06 |
| 12 | 67513611 | rs138129581 | T | 0,05 | 3,83 | 2,24 | 6,57 | 1,00E-06 |
| 21 | 18301110 | rs2824144 | A | 0,37 | 1,79 | 1,39 | 2,29 | 4,92E-06 |
| 21 | 18303318 | rs2243684 | A | 0,37 | 1,81 | 1,41 | 2,32 | 3,16E-06 |

N, number of participants; Chr, chromosome; SNP, single nucleotide polymorphism; OR, odds ratio; CI, confidence interval

**Table 2. Genome-wide suggestive level (*p* < 5 x 10^-6^) SNPs associated with painful TMD in female participants (N = 816) of NFBC 1966 study.**

| **Chr** | **Position** | **SNP** | **Effect allele** | **Effect allele frequency** | **OR** | **95% CI lower** | **95% CI upper** | **p-value** |
| --- | --- | --- | --- | --- | --- | --- | --- | --- |
| 2 | 8039194 | rs10929507 | T | 0,72 | 0,46 | 0,34 | 0,64 | 1,97E-06 |
| 2 | 168266061 | rs112436534 | T | 0,06 | 4,72 | 2,62 | 8,53 | 2,58E-07 |
| 2 | 168269170 | rs71428974 | G | 0,06 | 4,63 | 2,57 | 8,34 | 3,33E-07 |
| 2 | 168321539 | rs13026270 | G | 0,06 | 4,43 | 2,44 | 8,04 | 9,61E-07 |
| 2 | 193331208 | rs6741441 | C | 0,28 | 2,12 | 1,55 | 2,91 | 3,08E-06 |
| 2 | 193333903 | rs1385351 | T | 0,82 | 0,44 | 0,31 | 0,63 | 6,28E-06 |
| 2 | 193334433 | rs1385352 | A | 0,18 | 2,27 | 1,59 | 3,23 | 6,30E-06 |
| 2 | 193335659 | rs1484893 | T | 0,81 | 0,44 | 0,31 | 0,63 | 7,35E-06 |
| 2 | 193344575 | rs2357150 | C | 0,86 | 0,38 | 0,25 | 0,57 | 3,65E-06 |
| 2 | 193345479 | rs6713179 | G | 0,17 | 2,36 | 1,61 | 3,45 | 9,90E-06 |
| 2 | 193347387 | rs1471772 | G | 0,14 | 2,63 | 1,75 | 3,96 | 3,70E-06 |
| 2 | 193347925 | rs13432452 | C | 0,14 | 2,63 | 1,75 | 3,97 | 3,60E-06 |
| 2 | 193351161 | rs72913600 | T | 0,17 | 2,36 | 1,61 | 3,46 | 9,73E-06 |
| 2 | 196591273 | rs79514005 | C | 0,06 | 3,98 | 2,22 | 7,15 | 3,63E-06 |
| 2 | 196659034 | rs17442764 | A | 0,06 | 3,92 | 2,16 | 7,11 | 6,94E-06 |
| 2 | 196690296 | rs77036695 | T | 0,06 | 4,15 | 2,25 | 7,64 | 5,06E-06 |
| 2 | 196739009 | rs12469956 | A | 0,06 | 4,20 | 2,32 | 7,62 | 2,21E-06 |
| 2 | 230129269 | rs7600518 | G | 0,13 | 2,89 | 1,90 | 4,39 | 7,09E-07 |
| 2 | 230130271 | rs11898346 | C | 0,13 | 2,89 | 1,90 | 4,39 | 7,10E-07 |
| 2 | 230131445 | rs6754229 | C | 0,13 | 2,89 | 1,90 | 4,39 | 7,10E-07 |
| 2 | 230132158 | rs10185903 | C | 0,13 | 2,89 | 1,90 | 4,39 | 7,02E-07 |
| 2 | 230132239 | rs10186004 | G | 0,13 | 2,89 | 1,90 | 4,39 | 7,01E-07 |
| 2 | 230133054 | rs78366626 | G | 0,13 | 2,89 | 1,90 | 4,40 | 6,87E-07 |
| 2 | 230134014 | rs10180102 | C | 0,13 | 2,81 | 1,86 | 4,26 | 1,09E-06 |
| 2 | 230195686 | rs13387883 | A | 0,06 | 5,22 | 2,82 | 9,66 | 1,39E-07 |
| **2** | **230219445** | **rs148476652** | **C** | **0,05** | **6,95** | **3,64** | **13,25** | **4,01E-09** |
| 4 | 126722486 | rs4549391 | A | 0,62 | 1,98 | 1,48 | 2,65 | 4,21E-06 |
| 4 | 174739073 | rs12645518 | G | 0,38 | 1,92 | 1,44 | 2,55 | 8,13E-06 |
| 4 | 174746106 | rs4299559 | T | 0,41 | 1,89 | 1,43 | 2,50 | 8,33E-06 |
| 4 | 174746140 | rs9647500 | A | 0,41 | 1,89 | 1,43 | 2,50 | 8,33E-06 |
| 8 | 20567504 | rs2597401 | G | 0,87 | 0,39 | 0,26 | 0,59 | 6,03E-06 |
| 8 | 26027751 | rs17243976 | G | 0,08 | 3,22 | 1,96 | 5,30 | 4,14E-06 |
| 8 | 26029325 | rs77374642 | A | 0,08 | 3,21 | 1,94 | 5,30 | 5,29E-06 |
| 8 | 26030051 | rs79534917 | C | 0,08 | 3,21 | 1,94 | 5,30 | 5,26E-06 |
| 8 | 26030610 | rs55751400 | C | 0,08 | 3,21 | 1,94 | 5,30 | 5,23E-06 |
| 8 | 26038759 | rs17832905 | A | 0,06 | 4,62 | 2,48 | 8,59 | 1,39E-06 |
| 8 | 26042963 | rs78181362 | C | 0,08 | 3,25 | 1,97 | 5,37 | 4,09E-06 |
| 8 | 26043622 | rs77375686 | G | 0,08 | 3,25 | 1,97 | 5,37 | 4,08E-06 |
| 8 | 26043696 | rs111417100 | C | 0,08 | 3,25 | 1,97 | 5,37 | 4,08E-06 |
| 8 | 26046241 | rs56151795 | A | 0,08 | 3,25 | 1,97 | 5,38 | 4,06E-06 |
| 8 | 26046347 | rs55833772 | G | 0,08 | 3,25 | 1,97 | 5,38 | 4,05E-06 |
| 8 | 26047184 | rs77093188 | T | 0,08 | 3,25 | 1,97 | 5,36 | 4,21E-06 |
| 8 | 26049621 | rs28575061 | G | 0,08 | 3,25 | 1,97 | 5,37 | 4,11E-06 |
| 8 | 26050293 | rs79982991 | G | 0,08 | 3,26 | 1,97 | 5,38 | 4,03E-06 |
| 8 | 26051104 | rs80281594 | T | 0,08 | 3,26 | 1,97 | 5,38 | 4,03E-06 |
| 8 | 26060013 | rs13273938 | C | 0,08 | 3,06 | 1,87 | 5,02 | 9,41E-06 |
| 8 | 26060237 | rs13267151 | G | 0,08 | 3,06 | 1,87 | 5,02 | 9,40E-06 |
| 8 | 26061534 | rs13274883 | A | 0,08 | 3,06 | 1,87 | 5,02 | 9,40E-06 |
| 8 | 26062417 | rs71551853 | AC | 0,08 | 3,06 | 1,87 | 5,02 | 9,40E-06 |
| 8 | 26062870 | rs13268216 | C | 0,08 | 3,06 | 1,87 | 5,02 | 9,40E-06 |
| 8 | 26063734 | rs34788903 | C | 0,08 | 3,06 | 1,87 | 5,02 | 9,39E-06 |
| 8 | 41817704 | rs141641517 | C | 0,07 | 3,74 | 2,13 | 6,56 | 4,33E-06 |
| 8 | 77150358 | rs62515041 | A | 0,06 | 4,18 | 2,26 | 7,72 | 5,03E-06 |
| 8 | 77206236 | rs16939258 | T | 0,06 | 4,15 | 2,25 | 7,67 | 5,26E-06 |
| 8 | 77242013 | rs62515163 | C | 0,06 | 4,54 | 2,41 | 8,53 | 2,73E-06 |
| 8 | 78016748 | rs6473005 | T | 0,92 | 0,30 | 0,18 | 0,50 | 4,35E-06 |
| 8 | 78183590 | rs17374854 | A | 0,08 | 3,32 | 1,95 | 5,64 | 9,21E-06 |
| 8 | 78190714 | rs148278173 | T | 0,08 | 3,32 | 1,95 | 5,63 | 9,28E-06 |
| 8 | 78190719 | rs553088946 | G | 0,08 | 3,31 | 1,95 | 5,63 | 9,33E-06 |
| 8 | 78191123 | rs111623878 | C | 0,08 | 3,31 | 1,95 | 5,63 | 9,33E-06 |
| 8 | 78195302 | rs111513121 | T | 0,08 | 3,31 | 1,95 | 5,63 | 9,39E-06 |
| 8 | 78197851 | rs76629354 | A | 0,08 | 3,31 | 1,95 | 5,63 | 9,42E-06 |
| 8 | 78201019 | rs4073912 | A | 0,08 | 3,31 | 1,95 | 5,62 | 9,47E-06 |
| 8 | 78204359 | rs75244241 | T | 0,08 | 3,30 | 1,95 | 5,61 | 9,77E-06 |
| 9 | 79116755 | rs3739517 | C | 0,10 | 3,00 | 1,86 | 4,85 | 6,97E-06 |
| 12 | 9904449 | rs4763299 | T | 0,19 | 2,22 | 1,56 | 3,15 | 7,85E-06 |
| 12 | 9905271 | rs17806015 | G | 0,19 | 2,22 | 1,56 | 3,14 | 8,04E-06 |
| 12 | 9905948 | rs145524696 | C | 0,19 | 2,22 | 1,56 | 3,14 | 8,10E-06 |
| 12 | 9908429 | rs766502 | G | 0,19 | 2,21 | 1,56 | 3,14 | 8,32E-06 |
| 12 | 25057262 | rs3759088 | G | 0,72 | 0,49 | 0,36 | 0,67 | 9,62E-06 |
| 12 | 25058546 | rs4963818 | G | 0,72 | 0,49 | 0,36 | 0,67 | 9,85E-06 |
| 14 | 30775197 | rs76586945 | T | 0,08 | 3,11 | 1,90 | 5,07 | 5,65E-06 |
| 15 | 62425121 | rs17304101 | C | 0,09 | 3,30 | 1,96 | 5,55 | 7,42E-06 |
| 15 | 62425609 | rs201246185 | A | 0,09 | 3,30 | 1,96 | 5,55 | 7,42E-06 |
| 17 | 854348 | rs7216126 | G | 0,33 | 2,03 | 1,52 | 2,73 | 2,09E-06 |
| 17 | 72361381 | rs78028714 | T | 0,08 | 3,67 | 2,11 | 6,38 | 4,35E-06 |
| 18 | 2311131 | rs28641595 | T | 0,27 | 2,09 | 1,52 | 2,89 | 7,04E-06 |
| 20 | 8325287 | rs1232784 | C | 0,25 | 0,46 | 0,33 | 0,63 | 2,51E-06 |
| 20 | 8325562 | rs1234814 | T | 0,25 | 0,46 | 0,33 | 0,64 | 3,18E-06 |
| 20 | 8325569 | rs1775206 | G | 0,25 | 0,46 | 0,33 | 0,64 | 3,18E-06 |
| 20 | 8325978 | rs11908479 | T | 0,25 | 0,46 | 0,33 | 0,64 | 3,21E-06 |
| 20 | 8326177 | rs16994710 | C | 0,25 | 0,45 | 0,33 | 0,63 | 1,81E-06 |
| 20 | 8330515 | rs1232782 | G | 0,25 | 0,46 | 0,33 | 0,64 | 3,16E-06 |
| 20 | 8331955 | rs6086417 | A | 0,25 | 0,48 | 0,34 | 0,66 | 9,46E-06 |
| 20 | 8333841 | rs7354165 | G | 0,25 | 0,48 | 0,34 | 0,66 | 9,21E-06 |
| 20 | 8338567 | rs3031933 | GTAA | 0,27 | 0,44 | 0,32 | 0,61 | 7,44E-07 |
| 20 | 8348566 | rs708932 | G | 0,25 | 0,47 | 0,34 | 0,65 | 4,95E-06 |
| 22 | 46852430 | rs149971930 | T | 0,08 | 3,64 | 2,11 | 6,30 | 3,75E-06 |

N, number of participants; Chr, chromosome; SNP, single nucleotide polymorphism; OR, odds ratio; CI, confidence interval

**Table 3. Genome-wide suggestive level (*p* < 5 x 10^-6^) SNPs associated with painful TMD in male participants (N = 665) of NFBC 1966 study.**

| **Chr** | **Position** | **SNP** | **Effect allele** | **Effect allele frequency** | **OR** | **95% CI lower** | **95% CI**  **upper** | **p-value** |
| --- | --- | --- | --- | --- | --- | --- | --- | --- |
| 2 | 19018211 | rs1109635 | T | 0,06 | 10,15 | 3,95 | 26,09 | 1,52E-06 |
| 2 | 19018417 | rs1109634 | T | 0,06 | 10,14 | 3,94 | 26,08 | 1,52E-06 |
| 2 | 19020144 | rs57366408 | A | 0,06 | 10,12 | 3,94 | 26,00 | 1,54E-06 |
| 2 | 107761653 | rs150389455 | T | 0,12 | 5,07 | 2,54 | 10,12 | 4,25E-06 |
| 2 | 152216048 | rs28575212 | G | 0,17 | 3,95 | 2,20 | 7,07 | 3,87E-06 |
| 2 | 152217621 | rs62168616 | G | 0,17 | 3,95 | 2,20 | 7,07 | 3,87E-06 |
| 2 | 152217901 | rs59689616 | A | 0,17 | 3,95 | 2,20 | 7,07 | 3,87E-06 |
| 2 | 152218231 | rs3771892 | A | 0,17 | 3,91 | 2,18 | 7,00 | 4,43E-06 |
| 2 | 152218232 | rs3771893 | C | 0,17 | 3,95 | 2,21 | 7,08 | 3,78E-06 |
| 2 | 152221092 | rs3755480 | A | 0,17 | 3,95 | 2,20 | 7,07 | 3,86E-06 |
| 2 | 152224604 | rs3771897 | A | 0,17 | 3,95 | 2,20 | 7,07 | 3,86E-06 |
| 2 | 152225719 | rs77964389 | A | 0,13 | 4,63 | 2,41 | 8,90 | 4,30E-06 |
| 2 | 152227538 | rs10193875 | T | 0,17 | 3,95 | 2,20 | 7,07 | 3,85E-06 |
| 2 | 152227746 | rs16830005 | T | 0,17 | 3,95 | 2,20 | 7,07 | 3,85E-06 |
| 2 | 152228266 | rs13430270 | G | 0,17 | 3,95 | 2,20 | 7,07 | 3,85E-06 |
| 2 | 152228398 | rs13417340 | A | 0,17 | 3,95 | 2,21 | 7,07 | 3,81E-06 |
| 2 | 152229907 | rs58377527 | T | 0,17 | 3,95 | 2,20 | 7,07 | 3,85E-06 |
| 2 | 152230949 | rs6715069 | T | 0,17 | 3,95 | 2,20 | 7,07 | 3,85E-06 |
| 2 | 152231294 | rs3845844 | G | 0,17 | 3,95 | 2,20 | 7,07 | 3,85E-06 |
| 2 | 152232962 | rs62168620 | G | 0,17 | 3,91 | 2,19 | 6,99 | 4,25E-06 |
| 2 | 152233323 | rs6726714 | A | 0,17 | 3,95 | 2,20 | 7,07 | 3,86E-06 |
| 2 | 152233900 | rs4305272 | G | 0,17 | 3,95 | 2,20 | 7,07 | 3,85E-06 |
| 2 | 152234725 | rs11889610 | A | 0,13 | 4,69 | 2,44 | 9,01 | 3,74E-06 |
| 2 | 152235394 | rs62169366 | T | 0,13 | 4,68 | 2,43 | 9,01 | 3,75E-06 |
| 2 | 152235529 | rs113572081 | C | 0,17 | 3,95 | 2,20 | 7,07 | 3,85E-06 |
| 2 | 152236290 | rs1046672 | G | 0,17 | 3,96 | 2,21 | 7,09 | 3,65E-06 |
| 2 | 152236398 | rs1046675 | G | 0,17 | 3,95 | 2,20 | 7,07 | 3,85E-06 |
| 2 | 152240112 | rs5835364 | GTTTC | 0,21 | 3,80 | 2,18 | 6,64 | 2,70E-06 |
| 3 | 34704324 | rs113166906 | T | 0,07 | 8,78 | 3,59 | 21,44 | 1,89E-06 |
| 3 | 107691523 | rs113626048 | A | 0,16 | 5,06 | 2,71 | 9,46 | 3,75E-07 |
| 3 | 107692467 | rs709500 | G | 0,16 | 5,06 | 2,71 | 9,47 | 3,73E-07 |
| 3 | 107693015 | rs709499 | G | 0,16 | 5,06 | 2,71 | 9,47 | 3,73E-07 |
| 3 | 107693136 | rs840282 | G | 0,16 | 5,07 | 2,71 | 9,47 | 3,71E-07 |
| 5 | 108021870 | rs4957782 | C | 0,81 | 0,27 | 0,16 | 0,47 | 4,54E-06 |
| 5 | 108039280 | rs6887824 | A | 0,81 | 0,27 | 0,16 | 0,48 | 4,79E-06 |
| 5 | 108039636 | rs1533006 | G | 0,81 | 0,27 | 0,16 | 0,47 | 4,52E-06 |
| 5 | 111111113 | rs255889 | G | 0,08 | 7,32 | 3,23 | 16,60 | 1,92E-06 |
| 5 | 173171949 | rs7727718 | C | 0,11 | 6,84 | 3,25 | 14,39 | 4,08E-07 |
| 6 | 57162808 | rs1023114 | G | 0,07 | 14,40 | 5,90 | 35,19 | 4,82E-09 |
| 6 | 58196733 | rs145402506 | T | 0,05 | 15,59 | 5,51 | 44,12 | 2,29E-07 |
| 6 | 58330871 | rs139512125 | A | 0,06 | 13,50 | 4,85 | 37,57 | 6,17E-07 |
| 6 | 58465787 | rs149438017 | C | 0,05 | 13,57 | 4,69 | 39,23 | 1,48E-06 |
| 6 | 58473208 | rs202151411 | AT | 0,05 | 13,57 | 4,69 | 39,22 | 1,48E-06 |
| 6 | 58591086 | rs72870557 | G | 0,05 | 11,69 | 4,13 | 33,13 | 3,70E-06 |
| 7 | 109002435 | rs17134779 | C | 0,09 | 6,31 | 2,89 | 13,75 | 3,59E-06 |
| 8 | 20807635 | rs12545366 | G | 0,07 | 11,07 | 3,98 | 30,79 | 4,13E-06 |
| 10 | 120734125 | rs35082046 | TA | 0,90 | 0,14 | 0,06 | 0,31 | 1,01E-06 |
| 10 | 120735113 | rs945079 | C | 0,90 | 0,13 | 0,06 | 0,27 | 1,96E-07 |
| 12 | 34211314 | rs16921428 | A | 0,07 | 11,79 | 4,60 | 30,23 | 2,84E-07 |
| **12** | **34237012** | **rs148906547** | **A** | **0,07** | **13,05** | **5,17** | **32,95** | **5,44E-08** |
| 12 | 34251201 | rs56260627 | A | 0,07 | 11,98 | 4,66 | 30,80 | 2,52E-07 |
| **12** | **34357023** | **rs4244867** | **T** | **0,07** | **14,16** | **5,55** | **36,16** | **2,97E-08** |
| 12 | 34374327 | rs75988182 | A | 0,10 | 7,19 | 3,32 | 15,56 | 5,48E-07 |
| 12 | 34376890 | rs76905825 | G | 0,10 | 7,19 | 3,32 | 15,57 | 5,48E-07 |
| 12 | 34453488 | rs78434130 | G | 0,10 | 7,20 | 3,33 | 15,59 | 5,44E-07 |
| 12 | 34455052 | rs79909038 | A | 0,07 | 13,15 | 5,04 | 34,28 | 1,37E-07 |
| 12 | 34465363 | rs77254433 | T | 0,10 | 7,26 | 3,35 | 15,75 | 5,22E-07 |
| 12 | 34476201 | rs7397168 | C | 0,07 | 13,38 | 5,11 | 35,01 | 1,26E-07 |
| 12 | 34479072 | rs76921972 | A | 0,07 | 13,38 | 5,11 | 35,01 | 1,26E-07 |
| 12 | 133359914 | rs77055232 | T | 0,23 | 3,64 | 2,13 | 6,22 | 2,39E-06 |
| 12 | 133364785 | rs76848117 | T | 0,23 | 3,64 | 2,13 | 6,22 | 2,40E-06 |
| 12 | 133371541 | rs2873290 | A | 0,23 | 3,64 | 2,13 | 6,22 | 2,43E-06 |
| 12 | 133371546 | rs2316849 | A | 0,23 | 3,64 | 2,13 | 6,22 | 2,43E-06 |
| 12 | 133372923 | rs2242293 | T | 0,23 | 3,63 | 2,13 | 6,22 | 2,43E-06 |
| 12 | 133374682 | rs882378 | C | 0,23 | 3,61 | 2,11 | 6,18 | 2,72E-06 |
| 12 | 133374829 | rs882377 | A | 0,23 | 3,66 | 2,14 | 6,26 | 2,23E-06 |
| 12 | 133376387 | rs113213648 | T | 0,23 | 3,61 | 2,11 | 6,18 | 2,67E-06 |
| 12 | 133378610 | rs75002364 | A | 0,23 | 3,62 | 2,12 | 6,20 | 2,57E-06 |
| 12 | 133382934 | rs59417780 | A | 0,23 | 3,62 | 2,12 | 6,20 | 2,58E-06 |
| 12 | 133385626 | rs113986717 | A | 0,23 | 3,62 | 2,12 | 6,20 | 2,58E-06 |
| 12 | 133387385 | rs112781915 | T | 0,23 | 3,62 | 2,12 | 6,20 | 2,58E-06 |
| 12 | 133388460 | rs11147088 | T | 0,23 | 3,62 | 2,12 | 6,20 | 2,58E-06 |
| 12 | 133391022 | rs11836108 | A | 0,23 | 3,62 | 2,12 | 6,20 | 2,58E-06 |
| 12 | 133395038 | rs78719460 | A | 0,24 | 3,58 | 2,09 | 6,12 | 3,19E-06 |
| 12 | 133399553 | rs56362718 | C | 0,24 | 3,55 | 2,08 | 6,07 | 3,66E-06 |
| 12 | 133399746 | rs55960846 | T | 0,24 | 3,55 | 2,08 | 6,07 | 3,66E-06 |
| 12 | 133400246 | rs77000720 | A | 0,24 | 3,55 | 2,08 | 6,07 | 3,66E-06 |
| 12 | 133402391 | rs74552406 | G | 0,24 | 3,55 | 2,08 | 6,07 | 3,66E-06 |
| 12 | 133404805 | rs900448 | G | 0,24 | 3,55 | 2,08 | 6,07 | 3,67E-06 |
| 12 | 133405069 | rs3816760 | A | 0,24 | 3,55 | 2,08 | 6,07 | 3,67E-06 |
| 12 | 133411118 | rs725067 | C | 0,24 | 3,55 | 2,08 | 6,07 | 3,67E-06 |
| 12 | 133414054 | rs6560906 | C | 0,76 | 0,28 | 0,17 | 0,48 | 4,00E-06 |
| 12 | 133414560 | rs76934175 | A | 0,24 | 3,53 | 2,07 | 6,04 | 4,00E-06 |
| 12 | 133421213 | rs79216723 | C | 0,24 | 3,53 | 2,07 | 6,04 | 4,01E-06 |
| 12 | 133422157 | rs77719222 | A | 0,24 | 3,53 | 2,07 | 6,04 | 4,00E-06 |
| 12 | 133422367 | rs146400366 | GGGTGACTT | 0,24 | 3,53 | 2,07 | 6,04 | 4,00E-06 |
| 12 | 133426483 | rs11614340 | C | 0,24 | 3,53 | 2,07 | 6,04 | 4,01E-06 |
| 12 | 133428131 | rs2306540 | T | 0,24 | 3,53 | 2,07 | 6,04 | 4,01E-06 |
| 12 | 133432096 | rs56223638 | CA | 0,24 | 3,53 | 2,07 | 6,04 | 4,02E-06 |
| 12 | 133432439 | rs35290169 | G | 0,24 | 3,53 | 2,07 | 6,04 | 4,02E-06 |
| 12 | 133435186 | rs113796069 | T | 0,24 | 3,53 | 2,07 | 6,04 | 4,02E-06 |
| 12 | 133435716 | rs35011845 | G | 0,24 | 3,53 | 2,07 | 6,04 | 4,02E-06 |
| 12 | 133436866 | rs34182770 | C | 0,24 | 3,53 | 2,07 | 6,04 | 4,02E-06 |
| 12 | 133443005 | rs61625489 | C | 0,24 | 3,53 | 2,07 | 6,04 | 4,02E-06 |
| 12 | 133448391 | rs77936594 | A | 0,24 | 3,52 | 2,06 | 6,01 | 4,27E-06 |
| 12 | 133452069 | rs75830925 | T | 0,24 | 3,51 | 2,05 | 6,00 | 4,38E-06 |
| 12 | 133454567 | rs112898402 | G | 0,24 | 3,51 | 2,05 | 6,00 | 4,38E-06 |
| 12 | 133455883 | rs7957697 | G | 0,24 | 3,49 | 2,04 | 5,97 | 4,74E-06 |
| 12 | 133459973 | rs11608499 | C | 0,24 | 3,51 | 2,05 | 5,99 | 4,43E-06 |
| 12 | 133462182 | rs78901493 | A | 0,24 | 3,52 | 2,06 | 6,02 | 4,20E-06 |
| 12 | 133463086 | rs7136785 | A | 0,24 | 3,52 | 2,06 | 6,02 | 4,20E-06 |
| 12 | 133466869 | rs56325943 | T | 0,24 | 3,52 | 2,06 | 6,02 | 4,21E-06 |
| 12 | 133467284 | rs112191950 | A | 0,24 | 3,52 | 2,06 | 6,02 | 4,21E-06 |
| 12 | 133468232 | rs11608779 | T | 0,24 | 3,52 | 2,06 | 6,02 | 4,21E-06 |
| 12 | 133473809 | rs61649432 | T | 0,24 | 3,50 | 2,05 | 5,97 | 4,66E-06 |
| 12 | 133475690 | rs78092627 | A | 0,24 | 3,50 | 2,05 | 5,97 | 4,66E-06 |
| 12 | 133482500 | rs75563723 | A | 0,24 | 3,49 | 2,04 | 5,96 | 4,74E-06 |
| 12 | 133483582 | rs56864380 | C | 0,24 | 3,49 | 2,04 | 5,96 | 4,78E-06 |
| 13 | 24592365 | rs17349764 | A | 0,05 | 14,07 | 5,20 | 38,10 | 1,95E-07 |
| 14 | 85531518 | rs72681796 | C | 0,06 | 14,18 | 4,80 | 41,89 | 1,59E-06 |
| 16 | 69136777 | rs148876563 | C | 0,06 | 7,83 | 3,42 | 17,95 | 1,16E-06 |
| 16 | 69153251 | rs79051270 | T | 0,06 | 6,76 | 3,01 | 15,18 | 3,63E-06 |
| 16 | 69165972 | rs118052952 | A | 0,06 | 6,76 | 3,01 | 15,18 | 3,62E-06 |
| 16 | 69186927 | rs79663314 | A | 0,06 | 7,81 | 3,41 | 17,89 | 1,19E-06 |
| 16 | 69246614 | rs77041900 | T | 0,06 | 6,77 | 3,01 | 15,19 | 3,60E-06 |
| 16 | 69263471 | rs117028630 | A | 0,06 | 7,84 | 3,42 | 17,96 | 1,15E-06 |
| 16 | 69288723 | rs79650824 | A | 0,06 | 6,78 | 3,02 | 15,22 | 3,56E-06 |
| 16 | 69321201 | rs118090249 | A | 0,06 | 6,75 | 3,01 | 15,14 | 3,69E-06 |
| 16 | 69357406 | rs16958751 | A | 0,06 | 6,90 | 3,05 | 15,61 | 3,47E-06 |
| 16 | 69381286 | rs77901129 | T | 0,06 | 7,97 | 3,47 | 18,31 | 1,00E-06 |
| **16** | **69460794** | **rs74689594** | **C** | **0,05** | **10,28** | **4,44** | **23,81** | **5,42E-08** |
| 18 | 1144749 | rs138298731 | A | 0,05 | 16,66 | 5,88 | 47,22 | 1,21E-07 |
| 18 | 1153710 | rs79841648 | C | 0,06 | 20,90 | 7,57 | 57,68 | 4,42E-09 |
| 18 | 1205067 | rs150295012 | T | 0,08 | 7,86 | 3,27 | 18,88 | 3,96E-06 |
| 18 | 8479725 | rs149433130 | C | 0,10 | 6,60 | 3,19 | 13,66 | 3,74E-07 |
| 20 | 2361794 | rs16984767 | A | 0,14 | 4,70 | 2,43 | 9,11 | 4,34E-06 |
| 20 | 2366170 | rs56788947 | A | 0,14 | 4,76 | 2,46 | 9,22 | 3,66E-06 |
| 20 | 2366580 | rs6083018 | T | 0,14 | 4,77 | 2,46 | 9,23 | 3,61E-06 |
| 20 | 2373365 | rs73071383 | G | 0,14 | 4,72 | 2,44 | 9,12 | 3,82E-06 |
| 20 | 2422890 | rs76924566 | C | 0,08 | 7,74 | 3,46 | 17,32 | 6,33E-07 |

N, number of participants; Chr, chromosome; SNP, single nucleotide polymorphism; OR, odds ratio; CI, confidence interval

**Table 4. Genome-wide suggestive level (*p* < 5 x 10^-6^) SNPs associated with painful TMD in all participants (N = 5810) of Health 2000 survey.**

| **Chr** | **Position** | **SNP** | **Effect allele** | **Effect allele frequency** | **OR** | **95% CI lower** | **95% CI**  **upper** | **p-value** |
| --- | --- | --- | --- | --- | --- | --- | --- | --- |
| 2 | 29574781 | rs7573912 | T | 0,56 | 1,51 | 1,27 | 1,78 | 1,37E-06 |
| 2 | 79224138 | rs13427506 | G | 0,09 | 2,18 | 1,61 | 2,94 | 3,64E-07 |
| 2 | 1,6E+08 | rs72936417 | T | 0,06 | 2,36 | 1,68 | 3,33 | 8,13E-07 |
| 4 | 6434530 | rs34400800 | C | 0,42 | 1,48 | 1,25 | 1,75 | 3,84E-06 |
| 4 | 1,09E+08 | rs61357237 | T | 0,08 | 2,11 | 1,54 | 2,89 | 3,55E-06 |
| 8 | 95226924 | rs2623165 | T | 0,67 | 0,66 | 0,56 | 0,79 | 4,05E-06 |
| 8 | 1,16E+08 | rs78600969 | A | 0,06 | 2,46 | 1,68 | 3,59 | 3,57E-06 |
| 8 | 1,16E+08 | rs117434495 | T | 0,06 | 2,57 | 1,75 | 3,78 | 1,69E-06 |
| 14 | 26317882 | rs4983135 | T | 0,17 | 0,58 | 0,46 | 0,72 | 1,35E-06 |
| 16 | 88604957 | rs12446521 | T | 0,06 | 2,30 | 1,61 | 3,29 | 4,23E-06 |
| 20 | 19020751 | rs55865883 | T | 0,15 | 1,72 | 1,37 | 2,17 | 4,41E-06 |

N, number of participants; Chr, chromosome; SNP, single nucleotide polymorphism; OR, odds ratio; CI, confidence interval

**Table 5. Genome-wide suggestive level (*p* < 5 x 10^-6^) SNPs associated with painful TMD in female participants (N = 3172 ) of Health 2000 survey.**

| **Chr** | **Position** | **SNP** | **Effect allele** | **Effect allele frequency** | **OR** | **95%CI lower** | **95% CI**  **upper** | **p-value** |
| --- | --- | --- | --- | --- | --- | --- | --- | --- |
| 2 | 29574781 | rs7573912 | T | 0,56 | 1,58 | 1,30 | 1,91 | 2,80E-06 |
| 2 | 48953947 | rs76889437 | A | 0,20 | 0,54 | 0,43 | 0,69 | 8,59E-07 |
| 2 | 54915968 | rs10175711 | A | 0,09 | 2,14 | 1,53 | 2,98 | 7,19E-06 |
| 2 | 54916199 | rs13422098 | G | 0,09 | 2,13 | 1,53 | 2,97 | 7,63E-06 |
| 2 | 54916235 | rs13396897 | T | 0,09 | 2,13 | 1,53 | 2,97 | 7,67E-06 |
| 2 | 54916332 | rs7560775 | T | 0,09 | 2,13 | 1,53 | 2,98 | 7,59E-06 |
| 2 | 159858568 | rs72936417 | T | 0,06 | 2,46 | 1,66 | 3,65 | 8,30E-06 |
| 2 | 29574781 | rs7573912 | T | 0,56 | 1,58 | 1,30 | 1,91 | 2,80E-06 |
| 2 | 48953947 | rs76889437 | A | 0,20 | 0,54 | 0,43 | 0,69 | 8,59E-07 |
| 2 | 54915968 | rs10175711 | A | 0,09 | 2,14 | 1,53 | 2,98 | 7,19E-06 |
| 2 | 54916199 | rs13422098 | G | 0,09 | 2,13 | 1,53 | 2,97 | 7,63E-06 |
| 2 | 54916235 | rs13396897 | T | 0,09 | 2,13 | 1,53 | 2,97 | 7,67E-06 |
| 2 | 54916332 | rs7560775 | T | 0,09 | 2,13 | 1,53 | 2,98 | 7,59E-06 |
| 2 | 159858568 | rs72936417 | T | 0,06 | 2,46 | 1,66 | 3,65 | 8,30E-06 |
| 4 | 6434530 | rs34400800 | C | 0,42 | 1,58 | 1,30 | 1,91 | 2,80E-06 |
| 4 | 6434530 | rs34400800 | C | 0,42 | 1,58 | 1,30 | 1,91 | 2,80E-06 |
| 6 | 117689830 | rs1321805 | G | 0,14 | 1,92 | 1,44 | 2,56 | 8,96E-06 |
| 6 | 117689964 | rs142006240 | TA | 0,14 | 1,92 | 1,44 | 2,56 | 8,96E-06 |
| 6 | 117690638 | rs7738170 | T | 0,14 | 1,92 | 1,44 | 2,56 | 8,96E-06 |
| 6 | 117691070 | rs3798382 | A | 0,14 | 1,92 | 1,44 | 2,56 | 8,98E-06 |
| 6 | 117693515 | rs10434877 | G | 0,14 | 1,92 | 1,44 | 2,56 | 8,96E-06 |
| 6 | 117698419 | rs9374652 | A | 0,14 | 1,92 | 1,44 | 2,56 | 8,88E-06 |
| 6 | 117689830 | rs1321805 | G | 0,14 | 1,92 | 1,44 | 2,56 | 8,96E-06 |
| 6 | 117689964 | rs142006240 | TA | 0,14 | 1,92 | 1,44 | 2,56 | 8,96E-06 |
| 6 | 117690638 | rs7738170 | T | 0,14 | 1,92 | 1,44 | 2,56 | 8,96E-06 |
| 6 | 117691070 | rs3798382 | A | 0,14 | 1,92 | 1,44 | 2,56 | 8,98E-06 |
| 6 | 117693515 | rs10434877 | G | 0,14 | 1,92 | 1,44 | 2,56 | 8,96E-06 |
| 6 | 117698419 | rs9374652 | A | 0,14 | 1,92 | 1,44 | 2,56 | 8,88E-06 |
| 7 | 134590076 | rs2001277 | G | 0,08 | 2,26 | 1,58 | 3,24 | 9,62E-06 |
| 7 | 134605007 | . | T | 0,07 | 2,28 | 1,58 | 3,29 | 9,56E-06 |
| 7 | 134590076 | rs2001277 | G | 0,08 | 2,26 | 1,58 | 3,24 | 9,62E-06 |
| 7 | 134605007 | . | T | 0,07 | 2,28 | 1,58 | 3,29 | 9,56E-06 |
| 8 | 116184497 | rs116918532 | T | 0,06 | 2,73 | 1,75 | 4,25 | 9,01E-06 |
| 8 | 116200938 | rs78600969 | A | 0,06 | 2,71 | 1,74 | 4,22 | 9,61E-06 |
| 8 | 116242522 | rs117434495 | T | 0,06 | 2,95 | 1,88 | 4,64 | 2,49E-06 |
| 8 | 116184497 | rs116918532 | T | 0,06 | 2,73 | 1,75 | 4,25 | 9,01E-06 |
| 8 | 116200938 | rs78600969 | A | 0,06 | 2,71 | 1,74 | 4,22 | 9,61E-06 |
| 8 | 116242522 | rs117434495 | T | 0,06 | 2,95 | 1,88 | 4,64 | 2,49E-06 |
| 12 | 10241261 | rs117440358 | C | 0,12 | 2,19 | 1,62 | 2,96 | 3,47E-07 |
| 12 | 10251498 | rs148373579 | T | 0,08 | 2,24 | 1,57 | 3,19 | 8,18E-06 |
| 12 | 10241261 | rs117440358 | C | 0,12 | 2,19 | 1,62 | 2,96 | 3,47E-07 |
| 12 | 10251498 | rs148373579 | T | 0,08 | 2,24 | 1,57 | 3,19 | 8,18E-06 |
| 13 | 61406737 | rs3127050 | A | 0,71 | 1,65 | 1,33 | 2,04 | 3,94E-06 |
| 13 | 61407633 | rs3119870 | C | 0,71 | 1,65 | 1,33 | 2,04 | 3,83E-06 |
| 13 | 61407658 | rs3119871 | C | 0,71 | 1,65 | 1,33 | 2,04 | 3,80E-06 |
| 13 | 61407942 | rs3127051 | A | 0,71 | 1,65 | 1,33 | 2,04 | 3,94E-06 |
| 13 | 61406737 | rs3127050 | A | 0,71 | 1,65 | 1,33 | 2,04 | 3,94E-06 |
| 13 | 61407633 | rs3119870 | C | 0,71 | 1,65 | 1,33 | 2,04 | 3,83E-06 |
| 13 | 61407658 | rs3119871 | C | 0,71 | 1,65 | 1,33 | 2,04 | 3,80E-06 |
| 13 | 61407942 | rs3127051 | A | 0,71 | 1,65 | 1,33 | 2,04 | 3,94E-06 |
| 14 | 26297043 | rs8014315 | T | 0,33 | 0,61 | 0,50 | 0,76 | 4,31E-06 |
| 14 | 26301809 | rs61982219 | C | 0,33 | 0,63 | 0,51 | 0,77 | 9,88E-06 |
| 14 | 26305206 | rs72661402 | C | 0,33 | 0,63 | 0,51 | 0,77 | 9,59E-06 |
| 14 | 26308198 | rs142385273 | GATC | 0,33 | 0,63 | 0,51 | 0,77 | 8,30E-06 |
| 14 | 26308217 | rs61982220 | T | 0,31 | 0,61 | 0,49 | 0,75 | 2,20E-06 |
| 14 | 26317302 | rs17278195 | G | 0,31 | 0,61 | 0,49 | 0,75 | 2,30E-06 |
| 14 | 26297043 | rs8014315 | T | 0,33 | 0,61 | 0,50 | 0,76 | 4,31E-06 |
| 14 | 26301809 | rs61982219 | C | 0,33 | 0,63 | 0,51 | 0,77 | 9,88E-06 |
| 14 | 26305206 | rs72661402 | C | 0,33 | 0,63 | 0,51 | 0,77 | 9,59E-06 |
| 14 | 26308198 | rs142385273 | GATC | 0,33 | 0,63 | 0,51 | 0,77 | 8,30E-06 |
| 14 | 26308217 | rs61982220 | T | 0,31 | 0,61 | 0,49 | 0,75 | 2,20E-06 |
| 14 | 26317302 | rs17278195 | G | 0,31 | 0,61 | 0,49 | 0,75 | 2,30E-06 |
| 15 | 66420014 | rs16949541 | A | 0,32 | 1,69 | 1,36 | 2,10 | 2,09E-06 |
| 15 | 66420014 | rs16949541 | A | 0,32 | 1,69 | 1,36 | 2,10 | 2,09E-06 |
| 18 | 10364002 | rs206585 | C | 0,21 | 1,70 | 1,35 | 2,16 | 9,39E-06 |
| 18 | 10370771 | rs206580 | C | 0,22 | 1,69 | 1,34 | 2,12 | 6,84E-06 |
| 18 | 10364002 | rs206585 | C | 0,21 | 1,70 | 1,35 | 2,16 | 9,39E-06 |
| 18 | 10370771 | rs206580 | C | 0,22 | 1,69 | 1,34 | 2,12 | 6,84E-06 |

N, number of participants; Chr, chromosome; SNP, single nucleotide polymorphism; OR, odds ratio; CI, confidence interval

**Table 6. Genome-wide suggestive level (*p* < 5 x 10^-6^) SNPs associated with painful TMD in male participants (N = 2638 ) of Health 2000 survey.**

| **Chr** | **Position** | **SNP** | **Effect allele** | **Effect allele frequency** | **OR** | **95%CI lower** | **95% CI**  **upper** | **p-value** |
| --- | --- | --- | --- | --- | --- | --- | --- | --- |
| 1 | 22657679 | rs78252507 | G | 0,11 | 3,54 | 2,07 | 6,05 | 3,66E-06 |
| 1 | 78739478 | rs7550428 | G | 0,23 | 2,44 | 1,64 | 3,62 | 9,82E-06 |
| 1 | 78742185 | rs6685021 | T | 0,23 | 2,44 | 1,65 | 3,63 | 9,30E-06 |
| 1 | 78746576 | rs6658252 | C | 0,23 | 2,44 | 1,64 | 3,62 | 9,94E-06 |
| 1 | 95200019 | rs2640046 | T | 0,73 | 0,40 | 0,27 | 0,58 | 2,61E-06 |
| 1 | 96275380 | rs5776284 | G | 0,94 | 0,17 | 0,08 | 0,35 | 1,33E-06 |
| 1 | 96276165 | rs6678631 | C | 0,93 | 0,18 | 0,09 | 0,36 | 1,75E-06 |
| 1 | 96276219 | rs6677257 | C | 0,93 | 0,18 | 0,09 | 0,36 | 1,75E-06 |
| 1 | 96277018 | rs4494189 | A | 0,93 | 0,18 | 0,09 | 0,36 | 1,75E-06 |
| 1 | 96277166 | rs4511172 | G | 0,93 | 0,18 | 0,09 | 0,36 | 1,75E-06 |
| 1 | 96278122 | rs4950082 | C | 0,93 | 0,18 | 0,09 | 0,36 | 1,74E-06 |
| 1 | 96278438 | rs10158833 | G | 0,93 | 0,18 | 0,09 | 0,36 | 1,74E-06 |
| 1 | 96281108 | rs4494187 | A | 0,93 | 0,18 | 0,09 | 0,36 | 1,74E-06 |
| 1 | 96282357 | rs1935567 | T | 0,93 | 0,18 | 0,09 | 0,36 | 1,74E-06 |
| 1 | 96284284 | rs7552329 | T | 0,93 | 0,18 | 0,09 | 0,36 | 1,74E-06 |
| 1 | 96284631 | rs7514909 | C | 0,93 | 0,18 | 0,09 | 0,36 | 1,73E-06 |
| 1 | 96284667 | rs10159078 | G | 0,93 | 0,18 | 0,09 | 0,36 | 1,73E-06 |
| 1 | 96284980 | rs1342394 | A | 0,93 | 0,18 | 0,09 | 0,36 | 1,73E-06 |
| 1 | 96285367 | rs1342393 | T | 0,93 | 0,18 | 0,09 | 0,36 | 1,73E-06 |
| 1 | 96285376 | rs1342392 | C | 0,93 | 0,18 | 0,09 | 0,36 | 1,76E-06 |
| 1 | 96289924 | rs1361738 | G | 0,93 | 0,18 | 0,09 | 0,36 | 1,73E-06 |
| 1 | 96289936 | rs1361737 | C | 0,93 | 0,18 | 0,09 | 0,36 | 1,73E-06 |
| 1 | 96290423 | rs7545870 | C | 0,93 | 0,18 | 0,09 | 0,36 | 1,73E-06 |
| 1 | 96291755 | rs10874959 | G | 0,93 | 0,18 | 0,09 | 0,36 | 1,89E-06 |
| 1 | 96292836 | rs1418461 | T | 0,93 | 0,18 | 0,09 | 0,36 | 1,73E-06 |
| 1 | 96293388 | rs1418459 | C | 0,93 | 0,18 | 0,09 | 0,36 | 1,73E-06 |
| 1 | 96294141 | rs1539358 | A | 0,93 | 0,18 | 0,09 | 0,36 | 1,81E-06 |
| 1 | 96294754 | rs1418457 | G | 0,93 | 0,18 | 0,09 | 0,36 | 1,73E-06 |
| 1 | 96295015 | rs1418456 | T | 0,93 | 0,18 | 0,09 | 0,36 | 1,73E-06 |
| 1 | 96305433 | rs4950085 | A | 0,93 | 0,18 | 0,09 | 0,36 | 1,74E-06 |
| 1 | 96307778 | rs7514781 | A | 0,80 | 0,37 | 0,25 | 0,56 | 2,44E-06 |
| 1 | 96311691 | rs6663469 | T | 0,80 | 0,37 | 0,25 | 0,56 | 2,43E-06 |
| 1 | 96311940 | rs6673180 | G | 0,80 | 0,37 | 0,25 | 0,56 | 2,43E-06 |
| 1 | 96312166 | rs10783023 | G | 0,93 | 0,18 | 0,09 | 0,36 | 1,75E-06 |
| 1 | 96312644 | rs1418463 | C | 0,80 | 0,37 | 0,25 | 0,56 | 2,43E-06 |
| 1 | 96318346 | rs947642 | G | 0,80 | 0,37 | 0,25 | 0,56 | 2,42E-06 |
| 1 | 96320249 | rs1361741 | G | 0,93 | 0,18 | 0,09 | 0,36 | 1,79E-06 |
| 1 | 96321437 | rs6679428 | A | 0,79 | 0,39 | 0,26 | 0,59 | 5,95E-06 |
| 1 | 96321983 | rs7547144 | G | 0,79 | 0,39 | 0,26 | 0,59 | 5,99E-06 |
| 1 | 96325142 | rs34720455 | A | 0,79 | 0,39 | 0,26 | 0,59 | 6,26E-06 |
| 2 | 21805550 | rs144585772 | C | 0,05 | 6,97 | 2,98 | 16,30 | 7,48E-06 |
| 2 | 156216211 | rs114730203 | G | 0,06 | 5,42 | 2,57 | 11,44 | 9,39E-06 |
| 2 | 156299025 | rs79056312 | C | 0,06 | 5,40 | 2,56 | 11,39 | 9,57E-06 |
| 2 | 156434288 | rs17223913 | T | 0,08 | 4,21 | 2,24 | 7,93 | 8,19E-06 |
| 2 | 228884686 | rs61752226 | A | 0,06 | 5,52 | 2,60 | 11,71 | 8,52E-06 |
| 2 | 228932130 | rs115228891 | A | 0,06 | 5,52 | 2,60 | 11,71 | 8,43E-06 |
| 3 | 51101185 | rs78909740 | C | 0,06 | 5,88 | 2,73 | 12,69 | 6,26E-06 |
| 3 | 150566422 | rs13081927 | T | 0,13 | 3,46 | 2,02 | 5,92 | 5,74E-06 |
| 3 | 150570083 | rs13062669 | G | 0,13 | 3,51 | 2,07 | 5,98 | 3,51E-06 |
| 3 | 150570085 | rs13062671 | T | 0,13 | 3,51 | 2,07 | 5,98 | 3,51E-06 |
| 3 | 196373556 | rs73221626 | A | 0,08 | 4,95 | 2,55 | 9,61 | 2,38E-06 |
| 4 | 144640681 | rs62337585 | T | 0,07 | 5,19 | 2,52 | 10,71 | 8,24E-06 |
| 4 | 147779099 | rs1022074 | T | 0,26 | 2,41 | 1,65 | 3,52 | 5,22E-06 |
| 4 | 147779721 | rs6854020 | G | 0,26 | 2,41 | 1,65 | 3,52 | 5,25E-06 |
| 4 | 147779999 | rs6537434 | T | 0,26 | 2,41 | 1,65 | 3,53 | 5,20E-06 |
| 4 | 147784770 | rs6816827 | A | 0,26 | 2,41 | 1,65 | 3,53 | 5,20E-06 |
| 4 | 147802470 | rs34018903 | T | 0,26 | 2,47 | 1,69 | 3,61 | 3,18E-06 |
| 4 | 147815000 | rs141547815 | T | 0,26 | 2,47 | 1,69 | 3,62 | 3,15E-06 |
| 4 | 147818465 | rs34470581 | T | 0,26 | 2,47 | 1,69 | 3,61 | 3,21E-06 |
| 4 | 147820990 | rs984695 | C | 0,26 | 2,47 | 1,69 | 3,61 | 3,22E-06 |
| 4 | 147834157 | rs13138747 | A | 0,26 | 2,47 | 1,69 | 3,62 | 3,14E-06 |
| 4 | 147834758 | rs13122121 | C | 0,26 | 2,46 | 1,68 | 3,60 | 3,34E-06 |
| 4 | 147839474 | rs13106241 | C | 0,26 | 2,46 | 1,68 | 3,60 | 3,50E-06 |
| 4 | 147844562 | rs10519840 | C | 0,26 | 2,47 | 1,69 | 3,61 | 3,21E-06 |
| 4 | 147854082 | rs13121059 | T | 0,26 | 2,47 | 1,69 | 3,61 | 3,21E-06 |
| 4 | 147855158 | rs34431124 | T | 0,26 | 2,47 | 1,69 | 3,61 | 3,21E-06 |
| 4 | 147855939 | rs13130314 | T | 0,26 | 2,47 | 1,69 | 3,61 | 3,21E-06 |
| 4 | 147864614 | rs13104820 | T | 0,26 | 2,47 | 1,69 | 3,61 | 3,20E-06 |
| 4 | 147867307 | rs3827595 | C | 0,29 | 2,36 | 1,64 | 3,39 | 3,46E-06 |
| 4 | 147868430 | rs893231 | T | 0,29 | 2,36 | 1,64 | 3,39 | 3,43E-06 |
| 4 | 147868618 | rs62329360 | T | 0,29 | 2,36 | 1,64 | 3,39 | 3,43E-06 |
| 4 | 147869513 | rs882188 | G | 0,29 | 2,36 | 1,64 | 3,39 | 3,43E-06 |
| 4 | 147869725 | rs2884189 | A | 0,29 | 2,36 | 1,64 | 3,39 | 3,43E-06 |
| 4 | 147870570 | rs2004172 | T | 0,29 | 2,36 | 1,64 | 3,39 | 3,49E-06 |
| 4 | 147870746 | rs7658558 | C | 0,29 | 2,36 | 1,64 | 3,39 | 3,43E-06 |
| 4 | 147871005 | rs7680139 | A | 0,29 | 2,36 | 1,64 | 3,39 | 3,43E-06 |
| 4 | 147871058 | rs7664149 | C | 0,29 | 2,36 | 1,64 | 3,39 | 3,44E-06 |
| 4 | 147871271 | rs201027279 | A | 0,29 | 2,36 | 1,64 | 3,39 | 3,43E-06 |
| 4 | 147871276 | rs59404817 | A | 0,29 | 2,36 | 1,64 | 3,39 | 3,44E-06 |
| 4 | 147871511 | rs714627 | A | 0,29 | 2,36 | 1,64 | 3,39 | 3,43E-06 |
| 4 | 147872372 | rs141407303 | G | 0,29 | 2,36 | 1,64 | 3,39 | 3,43E-06 |
| 4 | 147872764 | rs969511 | G | 0,29 | 2,36 | 1,64 | 3,39 | 3,43E-06 |
| 4 | 147874266 | rs35712144 | T | 0,29 | 2,36 | 1,64 | 3,39 | 3,43E-06 |
| 4 | 147875027 | rs13120245 | G | 0,29 | 2,36 | 1,64 | 3,39 | 3,44E-06 |
| 4 | 147877183 | rs62329362 | G | 0,29 | 2,36 | 1,64 | 3,39 | 3,43E-06 |
| 4 | 147878112 | rs62329364 | G | 0,29 | 2,36 | 1,64 | 3,39 | 3,43E-06 |
| 4 | 147878581 | rs1437266 | G | 0,29 | 2,36 | 1,64 | 3,39 | 3,43E-06 |
| 4 | 147878632 | rs1437265 | T | 0,29 | 2,36 | 1,64 | 3,39 | 3,43E-06 |
| 4 | 147879993 | rs748828 | T | 0,29 | 2,36 | 1,64 | 3,39 | 3,43E-06 |
| 4 | 147884892 | rs34338807 | G | 0,29 | 2,36 | 1,64 | 3,39 | 3,43E-06 |
| 4 | 147885829 | rs67955354 | T | 0,29 | 2,36 | 1,64 | 3,39 | 3,42E-06 |
| 4 | 147887820 | rs6537437 | T | 0,29 | 2,36 | 1,64 | 3,39 | 3,42E-06 |
| 4 | 147888197 | rs7662306 | C | 0,29 | 2,36 | 1,64 | 3,39 | 3,42E-06 |
| 4 | 147888331 | rs7678787 | A | 0,29 | 2,36 | 1,64 | 3,39 | 3,42E-06 |
| 4 | 147889203 | rs56317226 | A | 0,29 | 2,36 | 1,64 | 3,39 | 3,41E-06 |
| 4 | 147890407 | rs34782747 | C | 0,29 | 2,36 | 1,64 | 3,39 | 3,39E-06 |
| 4 | 147890648 | rs993759 | C | 0,29 | 2,36 | 1,64 | 3,39 | 3,39E-06 |
| 4 | 147891155 | rs1437262 | G | 0,29 | 2,36 | 1,64 | 3,39 | 3,38E-06 |
| 4 | 147891683 | rs7670493 | G | 0,29 | 2,36 | 1,64 | 3,39 | 3,37E-06 |
| 4 | 147891793 | rs7669311 | A | 0,29 | 2,36 | 1,64 | 3,39 | 3,37E-06 |
| 4 | 147923166 | rs7660168 | C | 0,32 | 2,31 | 1,62 | 3,28 | 3,39E-06 |
| 4 | 147923616 | rs2357439 | C | 0,32 | 2,31 | 1,62 | 3,28 | 3,38E-06 |
| 4 | 147924404 | rs35334988 | C | 0,32 | 2,31 | 1,62 | 3,28 | 3,38E-06 |
| 4 | 147924603 | rs17472952 | G | 0,31 | 2,43 | 1,70 | 3,46 | 1,00E-06 |
| 4 | 147924767 | rs17472966 | T | 0,32 | 2,31 | 1,62 | 3,28 | 3,38E-06 |
| 4 | 147937092 | rs71592486 | AT | 0,32 | 2,32 | 1,63 | 3,30 | 3,08E-06 |
| 4 | 147937407 | rs34221140 | A | 0,32 | 2,32 | 1,63 | 3,30 | 2,95E-06 |
| 4 | 147937472 | rs56199987 | T | 0,32 | 2,32 | 1,63 | 3,30 | 2,95E-06 |
| 4 | 147939611 | rs13150187 | G | 0,32 | 2,32 | 1,63 | 3,30 | 3,09E-06 |
| 4 | 147941253 | rs6537445 | T | 0,36 | 2,25 | 1,59 | 3,17 | 3,91E-06 |
| 4 | 147943107 | rs7675515 | C | 0,32 | 2,32 | 1,63 | 3,30 | 3,03E-06 |
| 4 | 147947331 | rs3789147 | G | 0,32 | 2,32 | 1,63 | 3,30 | 3,01E-06 |
| 4 | 147950906 | rs867 | C | 0,32 | 2,32 | 1,63 | 3,31 | 2,89E-06 |
| 4 | 188067302 | rs28367566 | T | 0,07 | 4,80 | 2,55 | 9,04 | 1,14E-06 |
| 4 | 188069074 | rs12509151 | G | 0,07 | 4,80 | 2,55 | 9,04 | 1,14E-06 |
| 4 | 188072788 | c | A | 0,07 | 4,86 | 2,58 | 9,15 | 9,89E-07 |
| 4 | 188073028 | rs1532020 | C | 0,07 | 4,80 | 2,55 | 9,04 | 1,13E-06 |
| 4 | 188073463 | rs1532019 | G | 0,07 | 4,86 | 2,58 | 9,15 | 9,88E-07 |
| 4 | 188073479 | rs1532018 | T | 0,07 | 4,86 | 2,58 | 9,15 | 9,88E-07 |
| 4 | 188073853 | rs10016164 | A | 0,07 | 4,86 | 2,58 | 9,15 | 9,85E-07 |
| 4 | 188073901 | rs10016251 | C | 0,07 | 4,83 | 2,57 | 9,10 | 1,05E-06 |
| 4 | 188074033 | rs28571050 | T | 0,07 | 4,86 | 2,58 | 9,16 | 9,82E-07 |
| 4 | 188074906 | rs10019404 | A | 0,07 | 4,87 | 2,58 | 9,17 | 9,66E-07 |
| 4 | 188076519 | rs72719041 | C | 0,07 | 4,88 | 2,59 | 9,20 | 9,37E-07 |
| 4 | 188078932 | rs12506558 | A | 0,07 | 4,95 | 2,62 | 9,35 | 7,95E-07 |
| 4 | 188080313 | rs9996155 | G | 0,07 | 4,58 | 2,42 | 8,66 | 2,97E-06 |
| 4 | 188082904 | rs10550000 | C | 0,07 | 4,61 | 2,43 | 8,72 | 2,74E-06 |
| 5 | 83839479 | rs16901152 | G | 0,15 | 2,78 | 1,78 | 4,35 | 7,18E-06 |
| 5 | 83847507 | rs59889037 | T | 0,15 | 2,78 | 1,78 | 4,34 | 7,10E-06 |
| 6 | 115937397 | rs35036692 | C | 0,86 | 0,34 | 0,21 | 0,55 | 9,72E-06 |
| 6 | 115937730 | rs9400861 | T | 0,86 | 0,34 | 0,21 | 0,55 | 9,70E-06 |
| 6 | 115938997 | rs7774919 | A | 0,86 | 0,34 | 0,21 | 0,55 | 9,69E-06 |
| 6 | 115973931 | rs4946109 | T | 0,80 | 0,38 | 0,25 | 0,58 | 5,79E-06 |
| 6 | 116064559 | rs76828012 | C | 0,12 | 3,15 | 1,90 | 5,21 | 8,48E-06 |
| 6 | 116069733 | rs17077122 | G | 0,12 | 3,14 | 1,90 | 5,20 | 8,58E-06 |
| 6 | 116081396 | rs2882696 | C | 0,12 | 3,15 | 1,90 | 5,22 | 8,23E-06 |
| 6 | 162319341 | rs73022579 | G | 0,11 | 3,94 | 2,17 | 7,15 | 6,45E-06 |
| 7 | 88740523 | rs75029504 | A | 0,05 | 6,38 | 2,94 | 13,83 | 2,64E-06 |
| 7 | 88742607 | rs7810045 | T | 0,05 | 6,38 | 2,94 | 13,83 | 2,64E-06 |
| 7 | 88743045 | rs71557027 | C | 0,05 | 6,38 | 2,94 | 13,82 | 2,65E-06 |
| 7 | 88744851 | rs35887854 | C | 0,05 | 6,37 | 2,94 | 13,81 | 2,65E-06 |
| 7 | 88747870 | rs6942776 | T | 0,05 | 6,38 | 2,94 | 13,81 | 2,64E-06 |
| 7 | 88749160 | rs36065852 | A | 0,05 | 6,37 | 2,94 | 13,81 | 2,64E-06 |
| 7 | 88752793 | rs17166893 | A | 0,05 | 6,37 | 2,94 | 13,80 | 2,64E-06 |
| 7 | 88753829 | rs17166900 | T | 0,05 | 6,37 | 2,94 | 13,79 | 2,65E-06 |
| 7 | 88760129 | rs17166931 | T | 0,05 | 6,38 | 2,94 | 13,81 | 2,63E-06 |
| 7 | 88764256 | rs13246747 | A | 0,06 | 5,85 | 2,88 | 11,86 | 9,85E-07 |
| 7 | 88772405 | rs71557029 | C | 0,06 | 5,86 | 2,89 | 11,89 | 9,66E-07 |
| 7 | 88784820 | rs71557030 | G | 0,07 | 5,35 | 2,74 | 10,44 | 8,61E-07 |
| 7 | 88802283 | rs117967560 | C | 0,06 | 6,31 | 3,03 | 13,16 | 8,70E-07 |
| 7 | 124793437 | rs80299632 | G | 0,08 | 4,17 | 2,28 | 7,63 | 3,59E-06 |
| 7 | 129838400 | rs138275353 | A | 0,06 | 7,45 | 3,48 | 15,99 | 2,47E-07 |
| 8 | 1460750 | rs28412344 | A | 0,48 | 0,47 | 0,34 | 0,66 | 9,10E-06 |
| 10 | 73869663 | rs17726002 | A | 0,09 | 4,19 | 2,24 | 7,83 | 6,89E-06 |
| 11 | 10740549 | rs10840479 | A | 0,34 | 2,58 | 1,80 | 3,68 | 1,96E-07 |
| 11 | 23928672 | rs17234651 | A | 0,07 | 4,96 | 2,52 | 9,76 | 3,63E-06 |
| 11 | 111939975 | rs79404432 | A | 0,06 | 6,54 | 3,02 | 14,14 | 1,81E-06 |
| 11 | 111941959 | rs1425917 | A | 0,06 | 6,55 | 3,03 | 14,16 | 1,80E-06 |
| 12 | 10222356 | rs7136826 | T | 0,10 | 3,58 | 2,05 | 6,25 | 7,64E-06 |
| 12 | 121958610 | rs61953504 | G | 0,05 | 6,11 | 2,74 | 13,63 | 9,95E-06 |
| 13 | 32454282 | rs352 | C | 0,28 | 2,34 | 1,61 | 3,40 | 7,58E-06 |
| 15 | 53894755 | rs1906394 | A | 0,82 | 0,37 | 0,24 | 0,57 | 7,20E-06 |
| 15 | 63179781 | rs11637628 | C | 0,08 | 4,16 | 2,22 | 7,78 | 8,42E-06 |
| 15 | 63184678 | rs76586016 | G | 0,08 | 4,16 | 2,22 | 7,78 | 8,42E-06 |
| 15 | 63190182 | rs11639251 | A | 0,08 | 4,16 | 2,22 | 7,79 | 8,38E-06 |
| 15 | 63193151 | rs79789109 | A | 0,08 | 4,15 | 2,22 | 7,77 | 8,57E-06 |
| 15 | 63193330 | rs4775558 | G | 0,08 | 4,15 | 2,22 | 7,76 | 8,64E-06 |
| 15 | 63193412 | rs143760971 | T | 0,08 | 4,27 | 2,27 | 8,02 | 6,33E-06 |
| 15 | 63194693 | rs11636104 | T | 0,08 | 4,14 | 2,21 | 7,75 | 8,81E-06 |
| 15 | 63194872 | rs11636086 | T | 0,08 | 4,14 | 2,21 | 7,75 | 8,83E-06 |
| 15 | 63196232 | rs11633004 | C | 0,08 | 4,14 | 2,21 | 7,74 | 8,88E-06 |
| 15 | 63196792 | rs118135825 | A | 0,08 | 4,13 | 2,21 | 7,73 | 9,00E-06 |
| 15 | 63196896 | rs16946083 | G | 0,08 | 4,14 | 2,21 | 7,74 | 8,89E-06 |
| 15 | 63197552 | rs2029389 | T | 0,08 | 4,14 | 2,21 | 7,74 | 8,90E-06 |
| 15 | 63197833 | rs2414796 | C | 0,08 | 4,14 | 2,21 | 7,74 | 8,91E-06 |
| 15 | 63198244 | rs79289370 | C | 0,08 | 4,14 | 2,21 | 7,74 | 8,91E-06 |
| 15 | 63198461 | rs2414797 | T | 0,08 | 4,13 | 2,21 | 7,73 | 9,04E-06 |
| 15 | 63198517 | rs34825785 | T | 0,08 | 4,14 | 2,21 | 7,74 | 8,92E-06 |
| 15 | 63198563 | rs76355909 | C | 0,08 | 4,14 | 2,21 | 7,74 | 8,92E-06 |
| 15 | 63201582 | rs78335068 | A | 0,08 | 4,10 | 2,19 | 7,66 | 9,86E-06 |
| 15 | 63202946 | rs1489324 | T | 0,08 | 4,11 | 2,20 | 7,68 | 9,50E-06 |
| 15 | 63202955 | rs1489323 | C | 0,08 | 4,11 | 2,20 | 7,68 | 9,50E-06 |
| 15 | 63203068 | rs1489322 | A | 0,08 | 4,11 | 2,20 | 7,68 | 9,51E-06 |
| 15 | 63203237 | rs35933891 | C | 0,08 | 4,11 | 2,20 | 7,68 | 9,54E-06 |
| 15 | 63203484 | rs35235069 | G | 0,08 | 4,11 | 2,20 | 7,67 | 9,57E-06 |
| 15 | 63203751 | rs80000058 | A | 0,08 | 4,10 | 2,20 | 7,67 | 9,60E-06 |
| 15 | 63204274 | rs35102947 | T | 0,08 | 4,10 | 2,20 | 7,67 | 9,66E-06 |
| 15 | 63206440 | rs146089136 | G | 0,08 | 4,09 | 2,19 | 7,64 | 9,93E-06 |
| 15 | 63206779 | rs11630808 | C | 0,08 | 4,09 | 2,19 | 7,64 | 9,97E-06 |
| 15 | 63212912 | rs80112280 | T | 0,09 | 4,20 | 2,34 | 7,53 | 1,51E-06 |
| 15 | 70122166 | rs4777202 | G | 0,58 | 0,46 | 0,32 | 0,64 | 6,53E-06 |
| 15 | 70125489 | rs304986 | C | 0,49 | 0,44 | 0,32 | 0,62 | 2,85E-06 |
| 15 | 70126444 | rs11072122 | G | 0,50 | 2,15 | 1,53 | 3,02 | 8,90E-06 |

N, number of participants; Chr, chromosome; SNP, single nucleotide polymorphism; OR, odds ratio; CI, confidence interval

**Table 7. Genome-wide suggestive level (*p* < 5 x 10^-6^) SNPs associated with painful TMD in all participants (N = 7291 ) of NFBC 1966 study and Health 2000 survey meta-analysis.**

| **Chr** | **Position** | **SNP** | **Effect allele** | **Effect allele frequency** | **OR** | **95% CI lower** | **95% CI**  **upper** | **p-value** |
| --- | --- | --- | --- | --- | --- | --- | --- | --- |
| 1 | 152442159 | rs10564577 | T | 0.7 | 1.42 | 1.22 | 1.64 | 3.387e-06 |
| 2 | 159858568 | rs72936417 | T | 0.06 | 1.98 | 1.49 | 2.63 | 2.611e-06 |
| 3 | 3158068 | rs1669369 | T | 0.48 | 0.72 | 0.62 | 0.83 | 3.762e-06 |
| 3 | 3154893 | rs334792 | A | 0.52 | 1.39 | 1.21 | 1.6 | 4.139e-06 |
| 3 | 3157229 | rs1153461 | C | 0.52 | 1.39 | 1.21 | 1.6 | 4.403e-06 |
| 3 | 3152661 | rs7622183 | T | 0.53 | 0.72 | 0.63 | 0.83 | 4.407e-06 |
| 3 | 3157051 | rs1153462 | T | 0.48 | 0.72 | 0.63 | 0.83 | 4.461e-06 |
| 3 | 3156291 | rs2600027 | T | 0.48 | 0.72 | 0.63 | 0.83 | 4.537e-06 |
| 8 | 20600208 | rs1823471 | C | 0.08 | 1.83 | 1.41 | 2.37 | 4.397e-06 |
| 11 | 133737304 | rs1114367 | T | 0.5 | 0.73 | 0.64 | 0.83 | 4.63e-06 |
| 11 | 133749244 | rs10894757 | T | 0.5 | 1.37 | 1.2 | 1.57 | 4.733e-06 |
| 11 | 133737462 | rs10750551 | A | 0.5 | 0.73 | 0.64 | 0.84 | 4.848e-06 |
| 11 | 133739685 | rs10894755 | A | 0.5 | 1.37 | 1.2 | 1.57 | 4.922e-06 |
| 12 | 56539567 | rs11171756 | T | 0.82 | 0.65 | 0.54 | 0.78 | 3.214e-06 |
| 18 | 47069031 | rs9957889 | C | 0.74 | 1.45 | 1.24 | 1.69 | 2.471e-06 |
| 18 | 47064685 | rs8097491 | T | 0.74 | 1.44 | 1.24 | 1.68 | 2.816e-06 |
| 18 | 47067159 | rs17798781 | T | 0.26 | 0.69 | 0.6 | 0.81 | 2.964e-06 |
| 18 | 47072980 | rs55679522 | T | 0.74 | 1.44 | 1.24 | 1.68 | 3.174e-06 |
| 18 | 26703009 | rs16946236 | A | 0.24 | 1.44 | 1.23 | 1.68 | 3.911e-06 |

N, number of participants; Chr, chromosome; SNP, single nucleotide polymorphism; OR, odds ratio; CI, confidence interval

**Table 8. Genome-wide suggestive level (*p* < 5 x 10^-6^) SNPs associated with painful TMD in female participants (N = 3998) of NFBC 1966 study and Health 2000 survey meta-analysis.**

| **Chr** | **Position** | **SNP** | **Effect allele** | **Effect allele frequency** | **OR** | **95% CI lower** | **95% CI**  **upper** | **p-value** |
| --- | --- | --- | --- | --- | --- | --- | --- | --- |
| 1 | 238683538 | rs10495412 | A | 0.70 | 1.50 | 1.27 | 1.78 | 2.891e-06 |
| 7 | 71828223 | rs150852384 | A | 0.22 | 0.64 | 0.53 | 0.77 | 4.087e-06 |
| 7 | 71828564 | rs2968503 | A | 0.78 | 1.57 | 1.30 | 1.90 | 4.087e-06 |
| 7 | 71810805 | rs2968511 | C | 0.78 | 1.57 | 1.30 | 1.90 | 4.366e-06 |
| 12 | 221020 | rs11064535 | A | 0.06 | 2.47 | 1.68 | 3.65 | 4.801e-06 |
| 16 | 75544572 | rs80271939 | A | 0.94 | 0.44 | 0.31 | 0.62 | 2.027e-06 |
| 16 | 75542716 | rs117068603 | C | 0.94 | 0.44 | 0.31 | 0.62 | 2.062e-06 |
| 16 | 75544177 | rs148597897 | A | 0.06 | 2.28 | 1.62 | 3.20 | 2.11e-06 |

N, number of participants; Chr, chromosome; SNP, single nucleotide polymorphism; OR, odds ratio; CI, confidence interval

**Table 9. Genome-wide suggestive level (*p* < 5 x 10^-6^) SNPs associated with painful TMD in male participants (N = 3303) of NFBC 1966 study and Health 2000 survey meta-analysis.**

| **Chr** | **Position** | **SNP** | **Effect allele** | **Effect allele frequency** | **OR** | **95% CI lower** | **95% CI**  **upper** | **p-value** |
| --- | --- | --- | --- | --- | --- | --- | --- | --- |
| 2 | 241579108 | rs4676406 | T | 0.64 | 0.49 | 0.37 | 0.65 | 9.868e-07 |
| 2 | 70825729 | rs10193519 | T | 0.07 | 3.53 | 2.1 | 5.94 | 2.047e-06 |
| 2 | 241574401 | rs4676408 | A | 0.64 | 0.5 | 0.38 | 0.67 | 2.275e-06 |
| 2 | 70823702 | rs6741056 | T | 0.06 | 4 | 2.25 | 7.11 | 2.281e-06 |
| 2 | 70828494 | rs6739809 | A | 0.05 | 4.28 | 2.34 | 7.82 | 2.341e-06 |
| 4 | 101589209 | rs146664455 | A | 0.94 | 0.27 | 0.16 | 0.47 | 3.914e-06 |
| 6 | 162319341 | rs73022579 | A | 0.9 | 0.27 | 0.17 | 0.44 | 2.139e-07 |
| 8 | 138809298 | rs62530454 | A | 0.08 | 3.58 | 2.15 | 5.97 | 1.002e-06 |
| 8 | 40179515 | rs10958609 | T | 0.23 | 2.12 | 1.55 | 2.9 | 2.86e-06 |
| 9 | 101190754 | rs12378676 | T | 0.31 | 2.04 | 1.52 | 2.74 | 2.232e-06 |
| 9 | 101192981 | rs1572473 | A | 0.31 | 2.04 | 1.52 | 2.74 | 2.4e-06 |
| 9 | 101188911 | rs66989623 | A | 0.31 | 2.03 | 1.51 | 2.72 | 2.705e-06 |
| 9 | 101195899 | rs10986247 | A | 0.3 | 2.04 | 1.51 | 2.74 | 2.747e-06 |
| 9 | 101183344 | rs60868407 | T | 0.31 | 2.02 | 1.5 | 2.71 | 3.363e-06 |
| 17 | 76559802 | rs11652895 | T | 0.22 | 2.28 | 1.62 | 3.19 | 1.907e-06 |
| 17 | 76559529 | rs9907431 | A | 0.28 | 2.09 | 1.54 | 2.85 | 2.696e-06 |

N, number of participants; Chr, chromosome; SNP, single nucleotide polymorphism; OR, odds ratio; CI, confidence interval

**Figure 1. Manhattan plots for all participants of NFBC 1966 study discovery analysis (results of males and females are presented within the main text)**

**
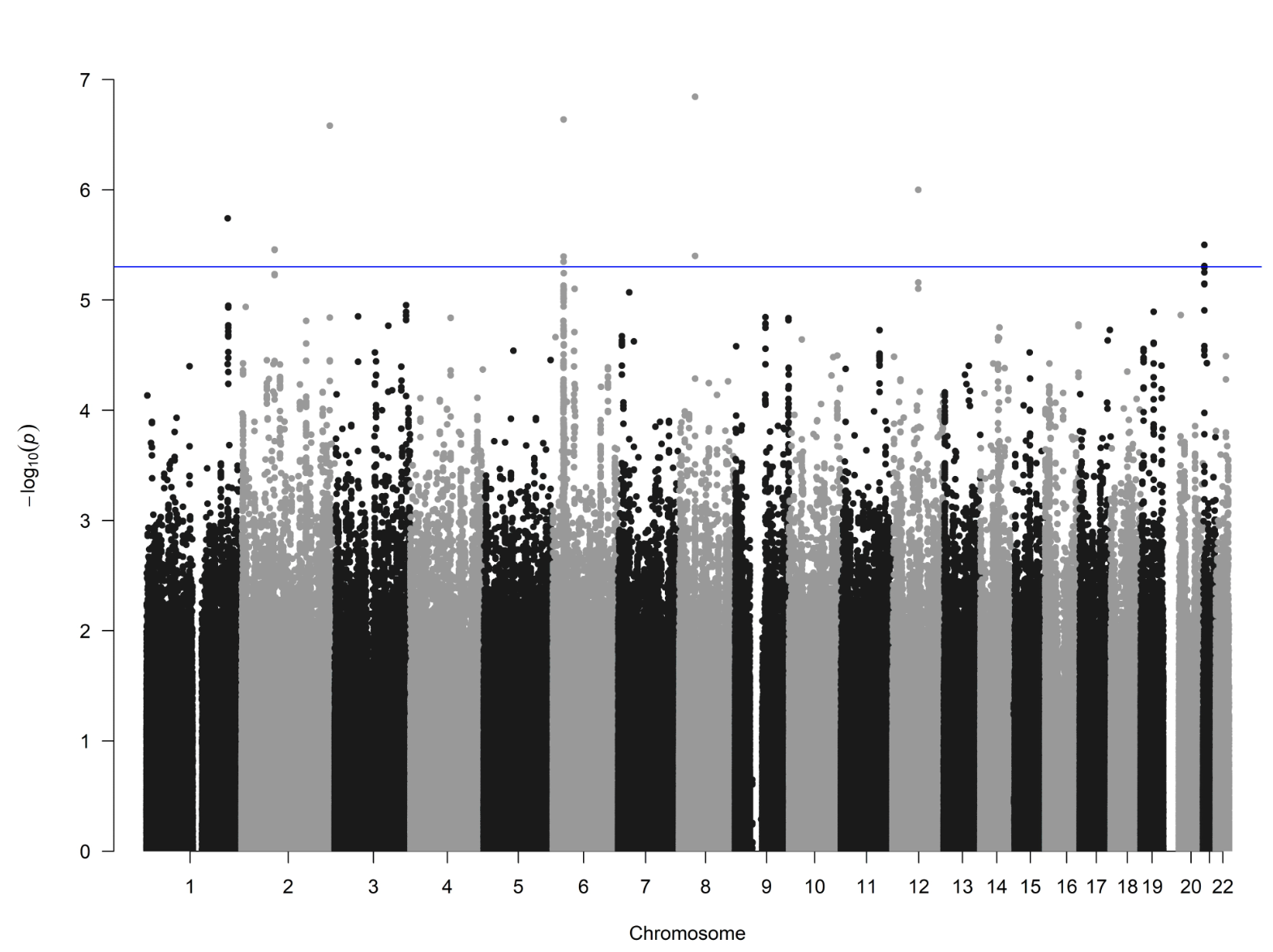
**

**Figure 2. Manhattan plots for A) all, B) female and C) male participants of Health 2000 survey discovery analysis**

**
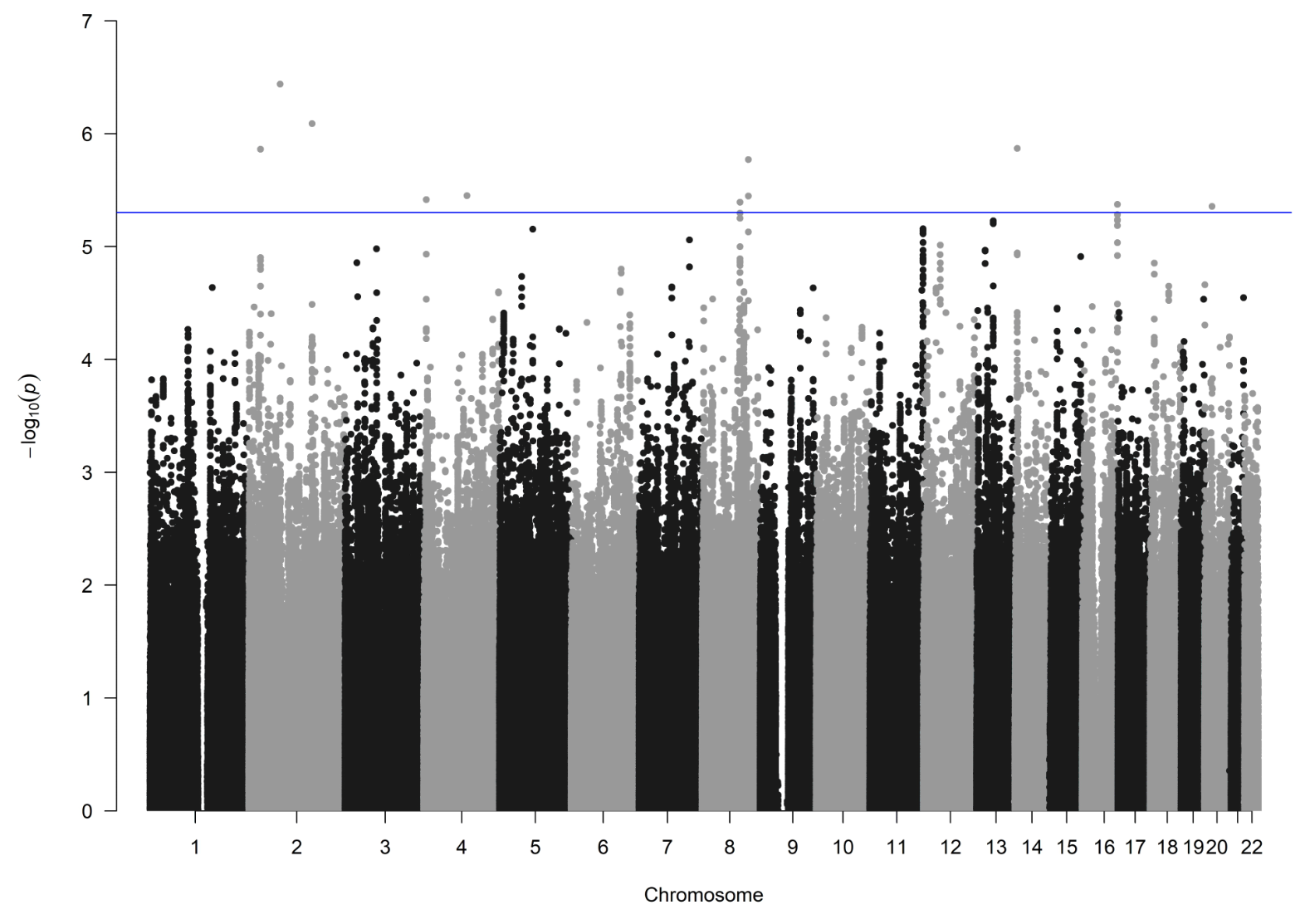
**

**B)**

**
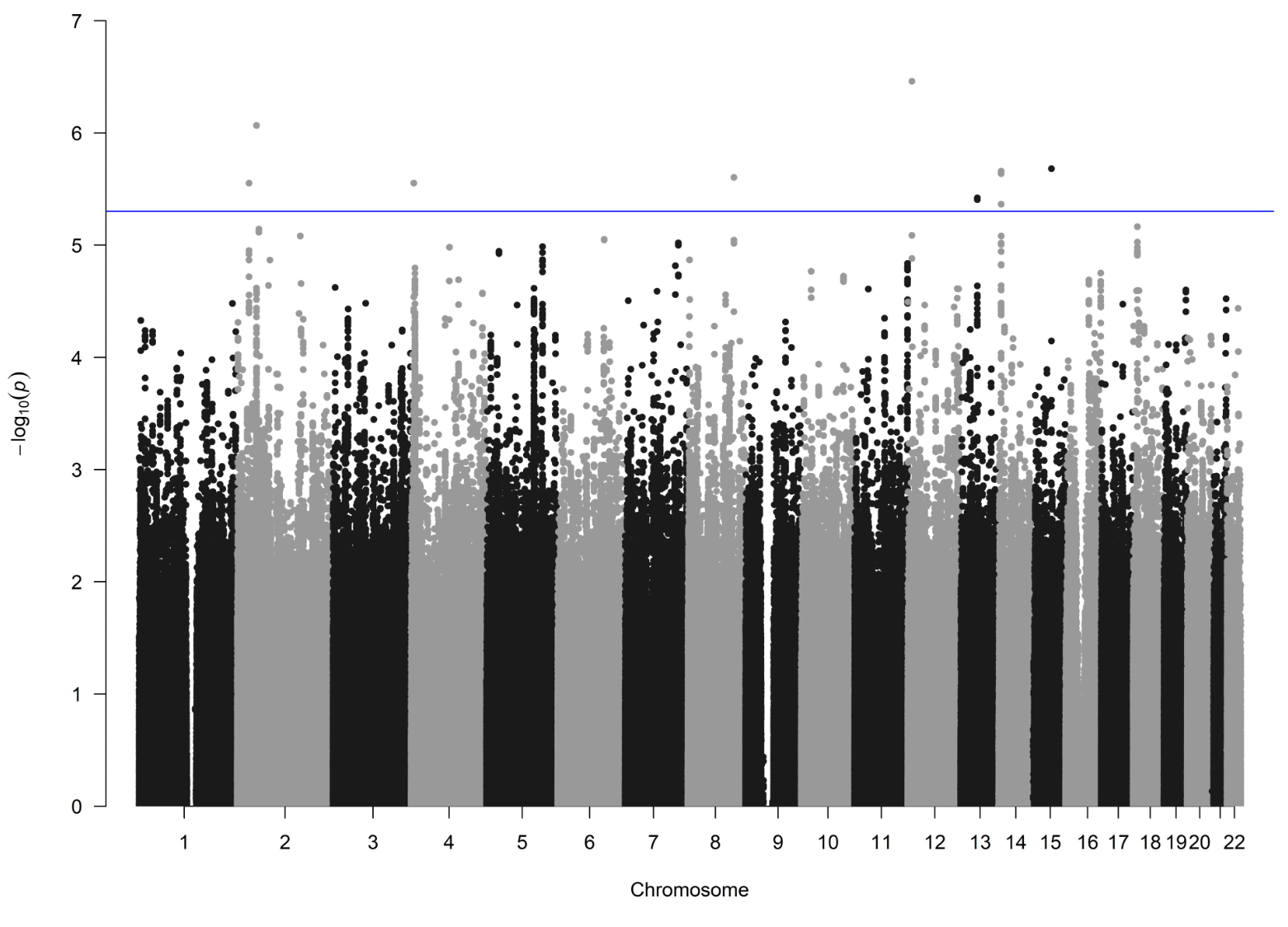
**

**C)**

**
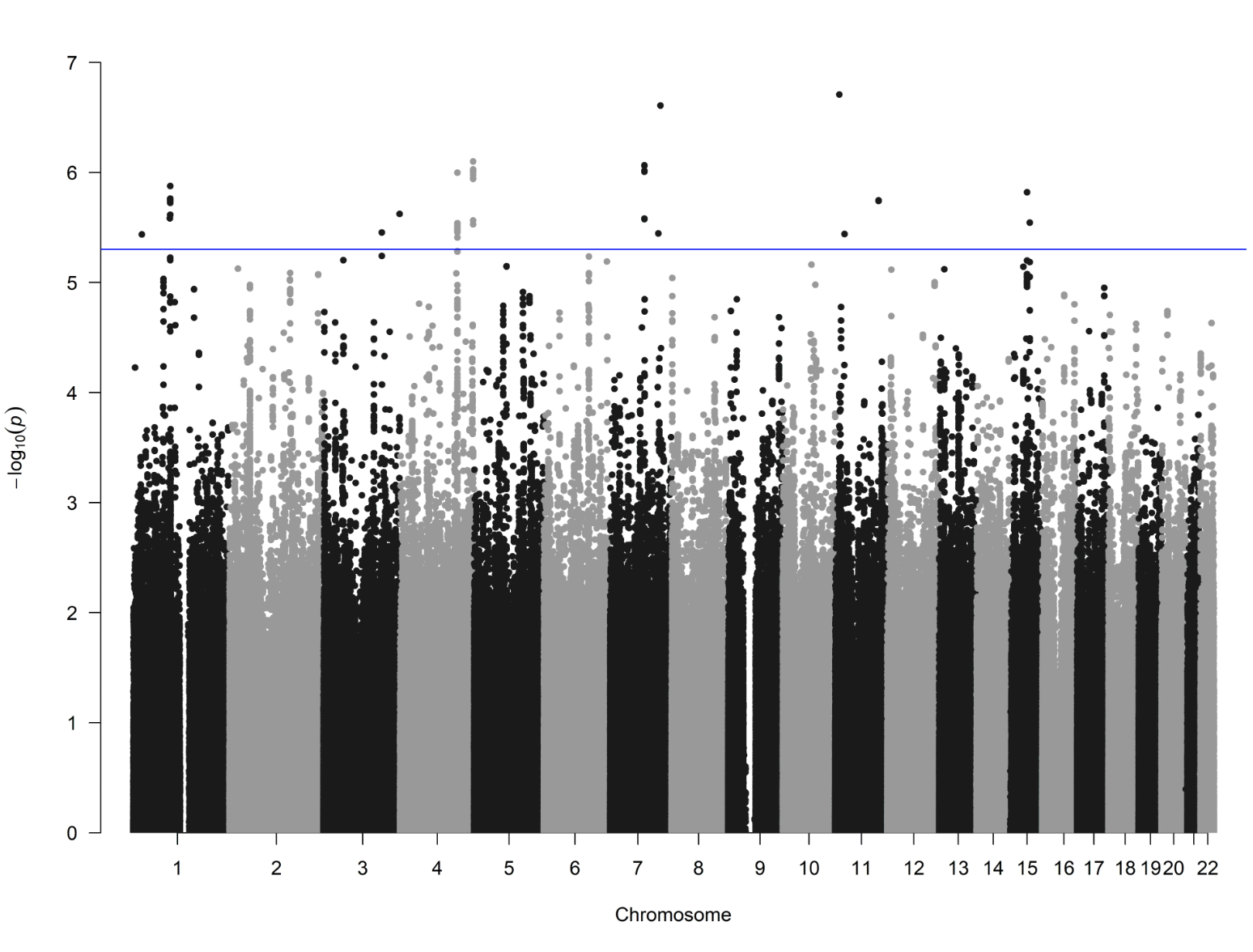
**

**Figure 3. Manhattan plots for A) all, B) female and C) male participants of NFBC 1966 study and Health 2000 survey meta-analysis.**

**A)**

**
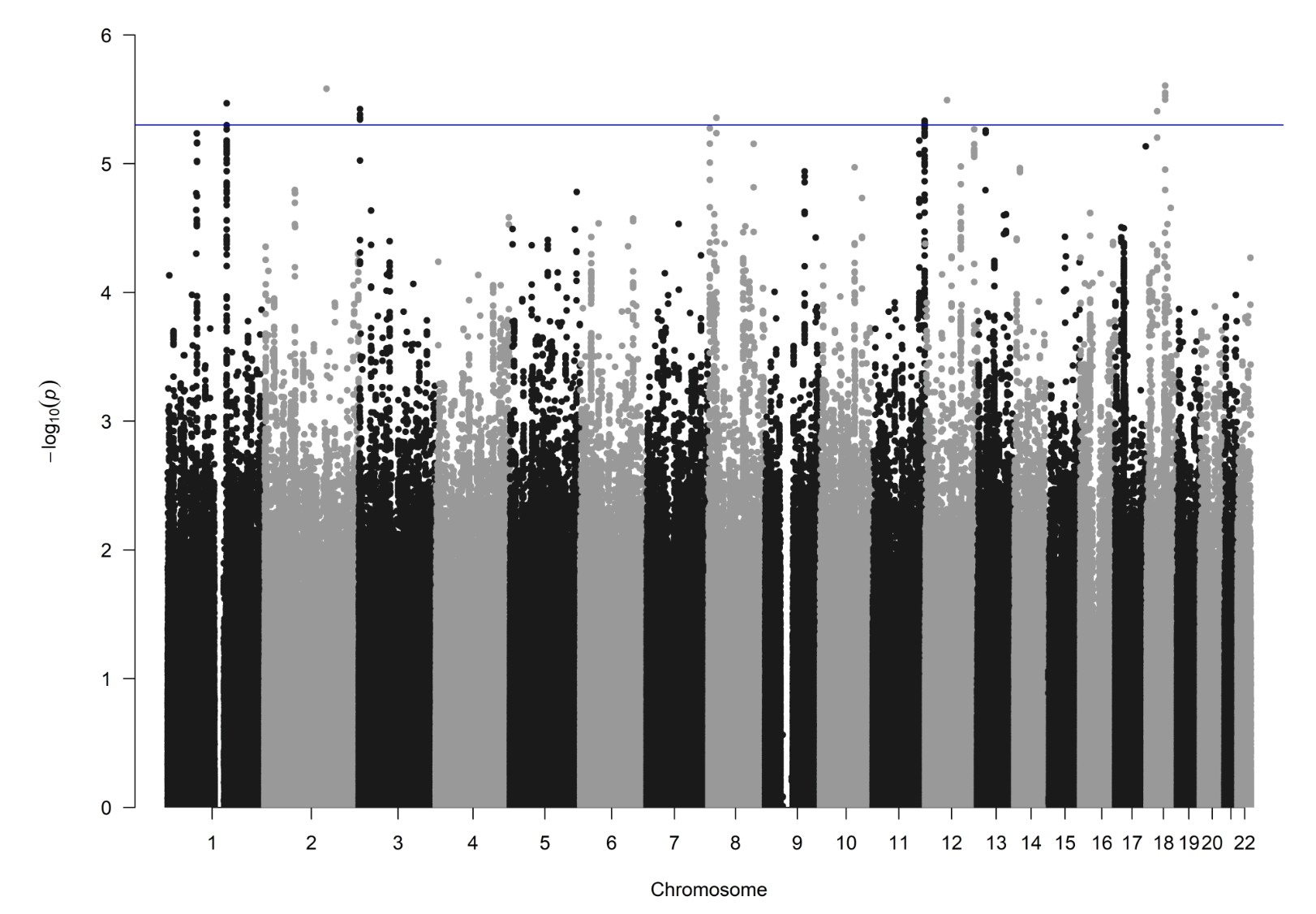
**

**B)**

**
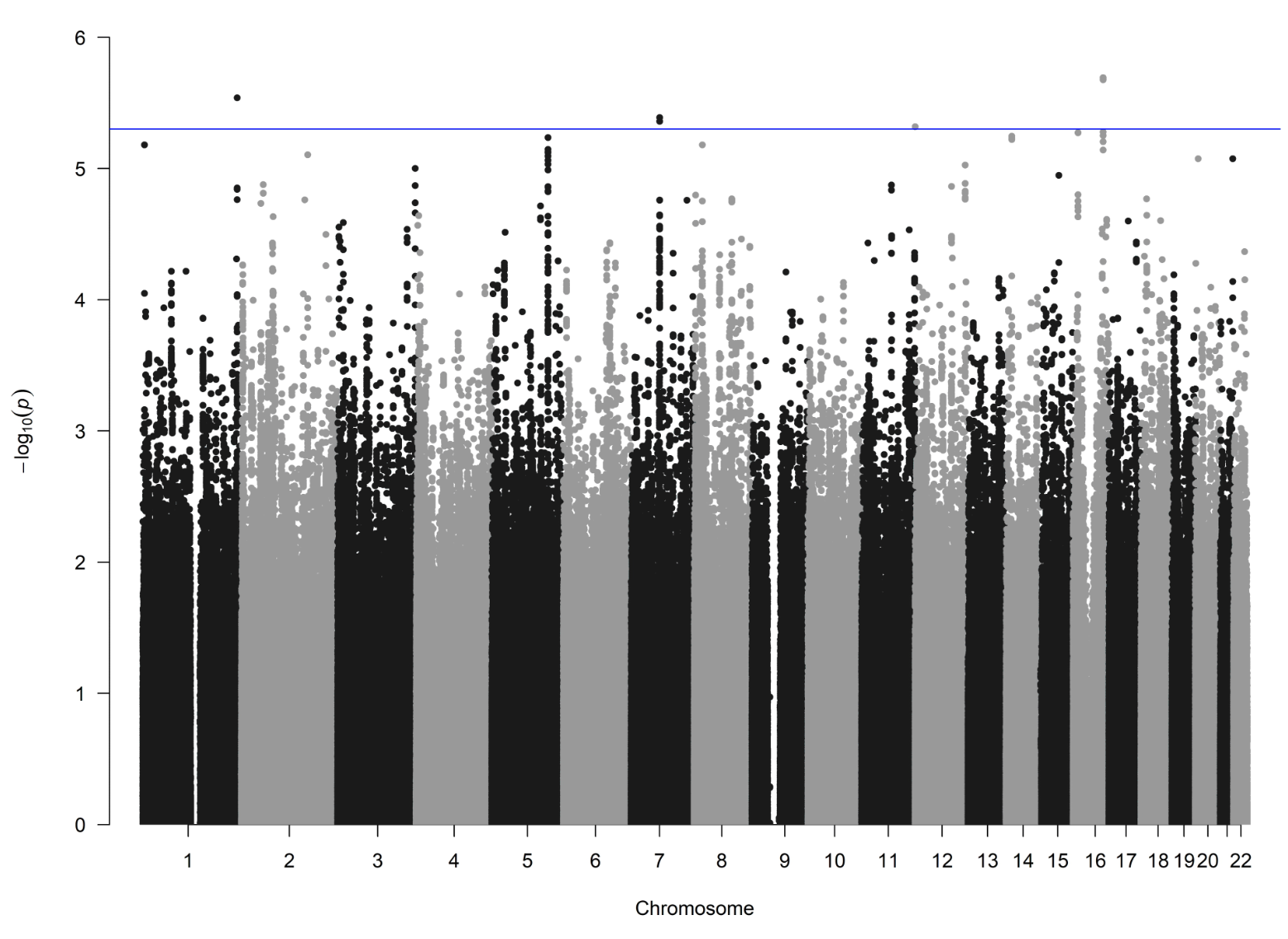
**

**C)**

**
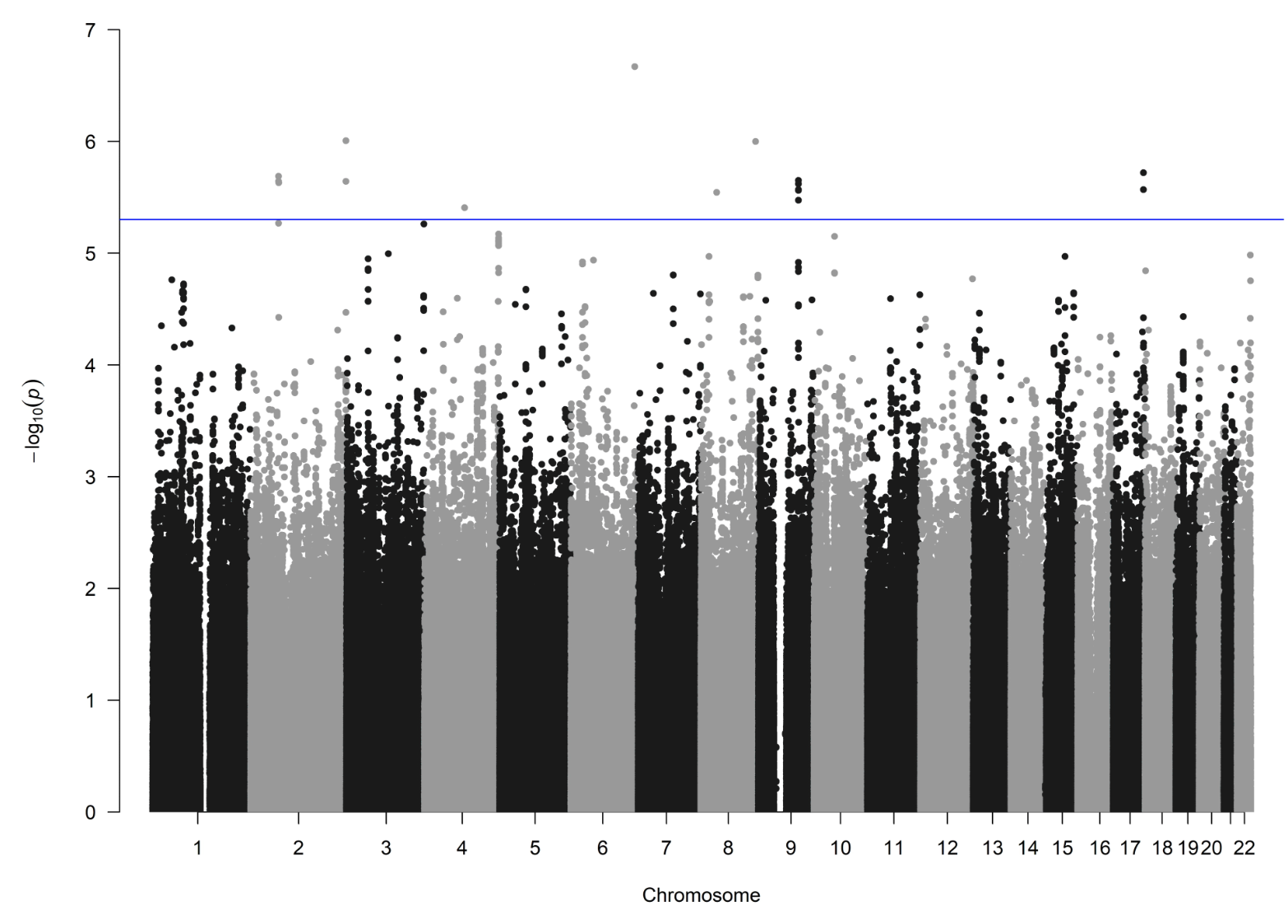
**

**Table 10 Replication analyses of the previously reported risk loci in TMD GWAS**

| Original GWAS | | | | | | |  | NFBC 1966 | | | | | |  | Health 2000 | | | | | |  | Meta-analysis of NFBC 1966 and Health 2000 | | | | | |
| --- | --- | --- | --- | --- | --- | --- | --- | --- | --- | --- | --- | --- | --- | --- | --- | --- | --- | --- | --- | --- | --- | --- | --- | --- | --- | --- | --- |
|  | Phenot. | Discovery | | | Replication Meta-analysis | |  | All | Female | | | Male | |  | All | | Female | | Male | |  | All | | Female | | Male | |
| Risk Loci in Previous GWAS | N  (sex str.) | Eff  All | OR  (95%CI) | P | OR  (95%CI) | P | Eff  All | OR  (95%CI) | P | OR  (95%CI) | P | OR  (95%CI) | P | Eff  All | OR  (95%CI) | P | OR  (95%CI) | P | OR  (95%CI) | P | Eff  All | OR  (95%CI) | P | OR  (95%CI) | P | OR  (95%CI) | P |
| *Sanders*  *et al (2017)*​^21^​ | Painful TMD |  |  |  |  |  |  |  |  |  |  |  |  |  |  |  |  |  |  |  |  |  |  |  |  |  |  |
| rs73460075 (Chr23, DMD) | 10142 (All) | G | 0.56 (NA) | 3.79  x 10^-8^ | NA | NA |  | NA | NA | NA | NA | NA | NA |  | NA | NA | NA | NA | NA | NA |  | NA | NA | NA | NA | NA | NA |
| rs4794106 (Chr17, SGCA) | 10153 (All) | T | 1.30 (NA) | 2.60  x 10^-6^ | 1.03  (0.94, 1.11) | 0.38 | C | 1.24 (0.98, 1.58) | 0.08 | 1.26  (0.95, 1.67) | 0.11 | 1.22  (0.77, 1.93) | 0.39 | C | 0.96  (0.81, 1.15) | 0.69 | 0.97  (0.79, 1.19) | 0.77 | 0.95  (0.66, 1.36) | 0.78 | T | 0.95  (0.82; 1.09) | 0.47 | 0.94  (0.80; 1.11) | 0.49 | 0,95  (0.72; 1.27) | 0.75 |
| rs73271865 (Chr7, SP4) | 10153 (All) | C | 0.56 (NA) | 2.91  x 10^-7^ | NA | NA | T | 1.01  (0.65, 1.56) | 0.98 | 1.12  (0.66, 1.89) | 0.68 | 0.76  (0.34, 1.69) | 0.50 | T | 0.90  (0.66, 1.22) | 0.48 | 0.78  (0.55, 1.11) | 0.17 | 1.37  (0.73, 2.56) | 0.33 | T | 0.93  (0.72; 1.19) | 0.58 | 0.88  (0.65; 1.18) | 0.37 | 1.09  (0.66;  1.79) | 0.74 |
| rs60249166 (Chr13, RXP2) | 5820 (Female) | C | 0.65 (NA) | 3.57  x 10^-8^ | 0.87  (0.74, 1.03) | 0.05 | T | 1.10  (0.83, 1.47) | 0.50 | 1.02  (0.72, 1.43) | 0.92 | 1.33  (0.77, 2.33) | 0.31 | T | 0.92  (0.74, 1.12) | 0.40 | 0.97  (0.76, 1.23) | 0.81 | 0.78  (0.51, 1.20) | 0.26 | T | 0.98  (0.82; 1.16) | 0.78 | 0.99  (0.81; 1.20) | 0.89 | 0.96  (0.68;  1.37) | 0.81 |
| rs1531554 (Chr17, BAHCCI) | 5820 (Female) | T | 0.68 (NA) | 2.92  x 10^-8^ | 0.83  (0.73, 0.95) | 0.002 | C | 0.85  (0.66, 1.09) | 0.20 | 0.81  (0.60, 1.08) | 0.15 | 1.02  (0.64, 1.64) | 0.93 | C | 1.11  (0.94, 1.32) | 0.21 | 1.16  (0.96, 1.41) | 0.12 | 0.95  (0.67, 1.35) | 0.79 | T | 0.98  (0.85; 1.12) | 0.74 | 0.96(  0.82; 1.13) | 0.63 | 1,02  (0.77;  1.36) | 0.88 |
| *Smith et al (2019)*​^22^​ | Painful TMD |  |  |  |  |  |  |  |  |  |  |  |  |  |  |  |  |  |  |  |  |  |  |  |  |  |  |
| rs5862730 (Chr4, OTUD4, SMAD1) | 3030 (All) | Del. | 1.42 (NA) | 2.82  x 10^-8^ | 1.00  (0.93, 1.07) | 0.48 | Del | 1.00  (0.78, 1.30) | 0.98 | 1.05  (0.77, 1.42) | 0.13 | 0.87  (0.54, 1.40) | 0.19 | Del | 1.02  (0.85,  1.22) | 0.85 | 1.01  (0.82, 1.24) | 0.95 | 1.06  (0.74, 1.53) | 0.75 | Del | 1.01  (0.87;  1.18) | 0.86 | 1.02  (0.86;  1.21) | 0.82 | 0.98 (0.74;  1.32) | 0.92 |
| rs10092633 (SFRP1) | 1956 (Female) | A | 4.12 (NA) | 2.91  x 10^-8^ | 0.89  (0.70, 1.13) | 0.33 |  | NA | NA | NA | NA | NA | NA |  | NA | NA |  | NA | NA | NA |  | NA | NA | NA | NA | NA | NA |
| rs34612513 (SOX14, CLDN18) | 1074 (Male) | A | 3.0 (NA) | 1.49  x 10^-8^ | 1.09  (0.94, 1.27) | 0.14 | A | 1.44 (0.89, 2.33) | 0.75 | 1.70  (0.96, 2.99) | 0.07 | 1.03  (0.40, 2.66) | 0.09 | A | 0.79  (0.55, 1.12) | 0.18 | 0.98  (0.66, 1.46) | 0.91 | 0.37  (0.18, 0.76) | **0.007** | A | 0.97 (0.73;  1.29) | 0.84 | 1.18 (0.85;  1.63) | 0.34 | 0.54  (0.30;  0.98) | **0.04** |
| *Yamaguchi et al.(2014)*​^26^​ | TMJ bony  degen. |  |  |  |  |  |  |  |  |  |  |  |  |  |  |  |  |  |  |  |  |  |  |  |  |  |  |
| rs878962 (TSPAN9) | 520  (All) | C | 1.94/1.78  (1.38,2.73/1.09,2.91) | NA | 1.89  (1.43, 2.50) | 8.2 x 10^-6^ | T | 1.19  (0.94, 1.51) | 0.14 | 1.06  (0.81, 1.41) | 0.65 | **1.67**  **(1.05, 2.63)** | **0.03** | T | 1.11  (0.94,  1.32) | 0.22 | 1.10  (0.91, 1.33) | 0.35 | 1.18  (0.84, 1.67) | 0.35 | T | 1.14  (0.99,  1.30) | 0.06 | 1.09  (0.93,  1.28) | 0.31 | **1.33 (1.01,**  **1.75)** | **0.04** |

N, number of participants; Chr, chromosome; SNP, single nucleotide polymorphism; OR, odds ratio; CI, confidence interval; P, p-value; NA, not available
